# Regium−π Bonds Involving Nucleobases: Theoretical Study and Biological Implications

**DOI:** 10.1021/acs.inorgchem.3c00369

**Published:** 2023-04-21

**Authors:** Sergi Burguera, Antonio Frontera, Antonio Bauza

**Affiliations:** Department of Chemistry, Universitat de les Illes Balears, Crta. de Valldemossa km 7.5, 07122 Palma, Baleares, Spain

## Abstract

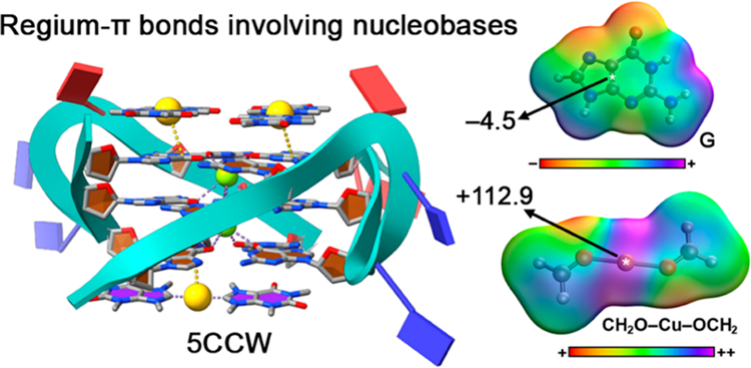

In this study, we provide crystallographic (Protein Data Bank (PDB) inspection) and theoretical (RI-MP2/def2-TZVP//PBE0-D3/def2-SVP level of theory) evidence of the involvement of nucleobases in Regium−π bonds (RgBs). This noncovalent interaction involves an electrophilic site located on an element of group 11 (Cu, Ag, and Au) and an electron-rich species (lone pair, LP donor, or π-system). Concretely, an initial PDB search revealed several examples where RgBs were undertaken involving DNA bases and Cu(II), Ag(I), and Au(I/III) ions. While coordination positions (mainly at the N atoms of the base) are well known, the noncovalent binding force between these counterparts has been scarcely studied in the literature. In this regard, computational models shed light on the strength and directionality properties of the interaction, which was also further characterized from a charge-density perspective using Bader’s “atoms in molecules” (AIM) theory, noncovalent interaction plot (NCIplot) visual index, and natural bonding orbital (NBO) analyses. As far as our knowledge extends, this is the first time that RgBs in metal–DNA complexes are systematically analyzed, and we believe the results might be useful for scientists working in the field of nucleic acid engineering and chemical biology as well as to increase the visibility of the interaction among the biological community.

## Abstract 2

Regium−π interactions involving nucleobases were studied at the RI-MP2/def2-TZVP level of theory in a combined crystallographic and theoretical study.

## Introduction

During the last decade, fast-growing revolution has taken place in the field of noncovalent interactions (NCIs), becoming essential resources of the chemist toolbox. They play a crucial role in several fields of modern chemistry, such as supramolecular chemistry,^1^ molecular recognition,^2^ and materials science.^3^ Despite the crucial role that hydrogen bonds (HBs) play in many chemical and biological environments,^4,5^ such as in enzyme catalysis and protein folding and binding phenomena (among others),^6^ a collection of NCIs based on the “σ-hole chemistry” (aerogen,^7^ halogen,^8^ chalcogen,^9^ pnictogen,^10^ and tetrel bonds)^11^ have emerged as novel and powerful resources for rational drug design,^12−14^ molecular aggregation,^15−17^ or even tuning self-assembly processes.^18−20^ A “σ-hole interaction” typically implies an electrophilic region from the σ-hole donor molecule (usually characterized by a positive electrostatic potential) located along the vector of a covalent bond (e.g., a C–Br bond in CBrF_3_ or an Sb–F bond in SbF_3_) that favorably interacts with a Lewis base (e.g., a lone pair, a π-system, or an anion).^21^

Recently, the “σ-hole” concept has been expanded to the transition-metal elements (d-block of the periodic table) through the discovery of several metal-based NCIs, such as Wolfium (group 6),^22^ Matere (group 7),^23^ Osme (group 8),^24^ Regium (group 11)^25,26^ and Spodium (group 12) bonds.^27,28^

In biology, metal complexes have been considered as a very powerful class of biological agents for decades. A typical example is represented by *cis*-diamminedichloridoplatinum(II) (cisplatin), which was considered the first clinically successful Pt anticancer drug; itself and various analogues being able to bind cellular DNA, stopping replication and inducing cell apoptosis.^29,30^ However, these compounds exhibited several disadvantages, such as limited solubility,^31^ severely dose-limiting side effects (e.g., nausea, neurotoxicity, and nephrotoxicity),^32,33^ and acquired resistance in some cancer variants.^34,35^ Hence, the development of novel compounds capable of disrupting cancerous cellular machinery by means of nonclassical interactions with nucleic acids has been the subject of study by many medicinal chemists.^36−40^ Also, this long-extended interest in metal–DNA interactions has resulted in the naissance of the field of DNA nanotechnology, where the incorporation of strongly bound metal cations promises to yield more robust, diversely functional DNA-based materials.^41−44^ The interaction between the metal complex and DNA is usually based on coordination chemistry, intercalation, or groove binding through an organic ligand portion.^45^ However, in this work, we are interested in the study of noncovalent metal···DNA interactions since they have been scarcely investigated.

In this regard, Regium−π interactions (Rg−π) were proposed a few years ago by some of us as an important and unnoticed supramolecular bond between group 11 elements (Cu, Ag, and Au) and aromatic systems,^26^ with potential applications in catalytic and organometallic chemistry processes.^46^ Very recently, we provided evidence of this interaction in biological systems by analyzing a series of protein–ligand interactions involving Cu, Ag, and Au in combined crystallographic and computational studies.^47,48^ However, no examples have been described so far in the literature involving Rg−π bonds in the field of metal complex–DNA/RNA interactions.

To that purpose, we started by performing a Protein Data Bank^49^ (PDB) survey of Cu(I/II), Ag(I), and Au(I/III) coordination complexes and manually inspected their direct interactions with nucleobases. This was complemented by a computational study at the RI-MP2/def2-TZVP//PBE0-D3/def2-SVP level of theory to analyze the physical nature and strength of the Rg−π bonds. Furthermore, state-of-the-art theoretical methodologies such as Bader’s quantum theory of atoms in molecules (QTAIM), noncovalent interaction plot (NCIplot) visual index and natural bonding orbital (NBO) analyses were used to further characterize the Rg−π complexes studied herein.

## Methods

### PDB Analysis

**Figure 1:**
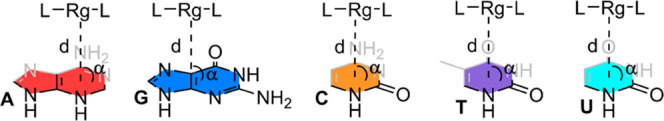
Schematic representation of the Rg−π complexes involving A, G, C, T, and U studied herein.

The PDB was manually inspected by focusing our attention on Cu(I/II), Ag(I), and Au(I/III) coordination complexes that were involved in Rg−π interactions. To classify the interaction as an Rg−π bond, the following geometrical criteria were used (see Figure 1):

1. The distance (*d*) Rg···nucleobase π-system was between 2.5 and 4 Å.
2. The angle (α, Rg···C_Nu_···C/N) was encompassed between 60 and 110°. The nucleobase centroid was defined on the six-membered ring in the case of A, C, T, and U and over the middle C–C double bond centroid in G.

## Complete List of PDB Codes

6M2P, 7BSE, 7BSF, 7ECL, 7SDH, 7SLJ, 7SMB, 2OIJ, 5CCW, 7EDV, and 2XY5.

### Computation of the Rg−π Bond Energies in Selected PDB Structures

Once the PDB structures were selected, theoretical models were built containing the cationic Rg coordination complex RgL_2_ (L = nucleobase) and the interacting nucleobase (either A, G, C, T, or U). The oxidation state of the Rg atom is included in Table 1. More in detail, the theoretical models of the Rg−π complexes from 7ECL, 7SDH, 7SMB, 2OJI, and 7EDV structures are composed of a Rg atom coordinated to two vicinal bases (A and U in 7ECL, C and C in 7SDH, U and U in 7SMB, and G and C in 2OJI and 7EDV). In 6M2P, a neutral cluster of 4 Ag atoms was used as a theoretical model of the Ag NP. Finally, in 5CCW, the Au(I) ion coordinated to two methylcaffein-8-ylidene molecules, and in 2XY5, the Cu(II) ion was coordinated to an organic ligand composed by two phenoxide groups and two imine groups. On the other hand, the π-system interacting with the Rg coordination complex involved a neutral nucleobase (adenine in 2XY5, 6M2P, 7ECL, 7SDH, and 7SMB, guanine in 2OIJ and 5CCW, and cytosine in 7EDV). See the SI for the Cartesian coordinates of the PDB models. In a later stage, the H atoms from the PDB models were optimized at the PBE0-D3/def2-SVP level of theory. These geometries were taken as the starting point for single-point calculations at the RI-MP2/def2-TZVP level of theory to compute the interaction energies given in Table 1.

**Table 1:** List of PDB Codes Retrieved from the Search (PDBID), Including the Interacting Partners (Rg Atom and Nucleobase), the BSSE-Corrected Energies (Δ*E*_BSSE_, in kcal/mol), Total Charge of the Rg−π Complex (Charge Rg−π), and Geometrical Parameters (*d*, in Å and α, in °) of Biological Rg−π Bonds

PDBID	Rg atom	nucleobase	charge Rg−π	Δ*E*_BSSE_	*d*^a^	α^b^
2XY5	Cu(II)	A	0	–7.4	3.592	74.3
6M2P	Ag(0)	A	0	–12.4	3.282	78.0
7ECL	Ag(I)	A	0	–6.9	2.832	75.9
7SDH	Ag(I)	A	+1	–20.4	2.807	82.2
7SMB	Ag(I)	A	0	–5.5	3.488	82.6
2OIJ	Au(III)	G	+2	–33.8	3.167	88.6
5CCW	Au(I)	G	+1	–14.3	3.514	86.2
7EDV	Au(I)	C	+1	–14.7	3.281	74.2

a Values given using the shortest Rg···π-system distance.

b Values given as the Rg···ring centroid···C/N for adenine and cytosine complexes. In the case of A and C, the centroid was placed on the six-membered ring. In the case of G, a central C–C double bond centroid was used.

### Computation of the Rg−π Bond Energies Using Optimized Models (Complexes **1**–**30**)

The interaction energies of all complexes included in this study were computed at the RI-MP2^50^/def2-TZVP^51^ level of theory. The calculations were performed using the program TURBOMOLE version 7.0.^52^ During the starting phase, a distance scan (involving the Rg···C_Nu_ (C_Nu_ = centroid nucleobase) distance) was performed at the PBE0^53,54^-D3^55^/def2-SVP^51^ level of theory on complexes **1**–**30**. Using the most stable point along the scanned coordinate, the complex was relaxed while keeping the Rg···C_Nu_ distance frozen at the RI-MP2/def2-TZVP level of theory to finally obtain the values shown in the energetic study (see below). The binding energies were calculated using the supermolecule approximation (Δ*E*_complex_ = *E*_complex_ – *E*_monomer1_ – *E*_monomer2_). Relaxation of the system without constraining the Rg···C_Nu_ distance led to a different geometry, marked by a coordination bond between the metal center and the O/N atoms of the nucleobase with much larger interaction energies. Since the optimized structures do not correspond to fully relaxed geometries, frequency analysis calculations were not performed.

The molecular electrostatic potential (MEP) surfaces were computed at the RI-MP2/def2-TZVP level of theory by means of the TURBOMOLE 7.0 program and analyzed using Multiwfn software.^56^ The calculations for the wavefunction analysis were carried out at the RI-MP2/def2-TZVP level of theory, also using Multiwfn software. The NBO analyses were performed at the HF/def2-TZVP level of theory by means of the NBO 7.0 program.^57^ Lastly, the NCIplot^58^ isosurfaces correspond to both favorable and unfavorable interactions, as differentiated by the sign of the second-density Hessian eigenvalue and defined by the isosurface color. The color scheme is a red–yellow–green–blue scale, with red for repulsive (ρ_cut_^+^) and blue for attractive (ρ_cut_^–^) NCI interaction density. Yellow and green surfaces correspond to weak repulsive and weak attractive interactions, respectively.

## Results and Discussion

### PDB Survey

**Figure 2:**
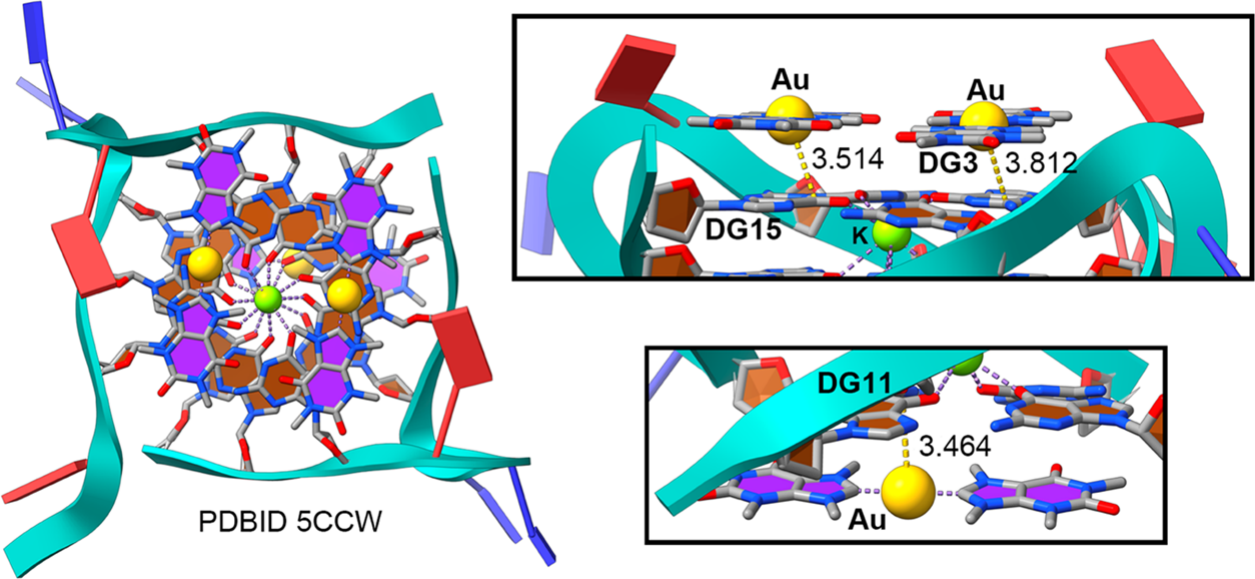
Regium−π interactions in the crystal structure 5CCW. The interactions are magnified inside the rectangular parts of the figure. Yellow dashed lines indicate Au···Guanine Rg−π bonds in Å. Purple dashed lines indicate coordination bonds.

We started by inspecting the Protein Data Bank in order to find crystallographic evidence of the presence of Rg−π bonds involving nucleobases (see the Methods section for specific details). A total number of 11 structures were found involving Cu/Ag/Au Rg−π bonds with nucleobases (A, G, and C). Three representative structures involving G and A rings derived from the search were selected and discussed in this section. In the first example (Figure 2), Bazzicalupi and collaborators^59^ used a combination of electron spray ionization-mass spectrometry (ESI MS) and X-ray diffraction (XRD) techniques to solve the crystal structure of a dicarbene Au(I) complex bound to a telomeric DNA G-Quadruplex (PDBID: 5CCW). Briefly explained, G-quadruplexes are nucleic acid sequences rich in guanines, where four guanine bases are bound through Hoogsteen HBs, forming a square-planar motif, named “guanine tetrad” and two or more guanine tetrads stack on top of each other to form a G-quadruplex.^60^ The quadruplex structure is further stabilized by the presence of monovalent cations, which lie in the central channel between each pair of tetrads^61^ (K^+^ in 5CCW structure). Interestingly, the formation of quadruplexes causes a net decrease in the activity of the enzyme telomerase, whose main function is the maintenance of the telomeres’ length;^62^ thus, the development of efficient quadruplex DNA binders is of great interest in the field of chemical biology and pharmacology. In their study, the authors pointed out the noncovalent character of the cationic interaction between the Au(I) dicarbene complex and the π-system of G, a specific contact that contributed to the binding affinity towards the quadruplex unit. As noticed in Figure 2, several Rg−π contacts are established between these two counterparts, involving the π-systems of G15, G3, and G11. This can be anticipated by the intermolecular distances observed (between 3.4 and 3.9 Å), which are larger than a classical coordination bond. We computed the interaction strength for the Rg−π contact involving G15, resulting in −14.3 kcal/mol, which clearly differs from a classical Au–C coordination bond energy.^63^

**Figure 3:**
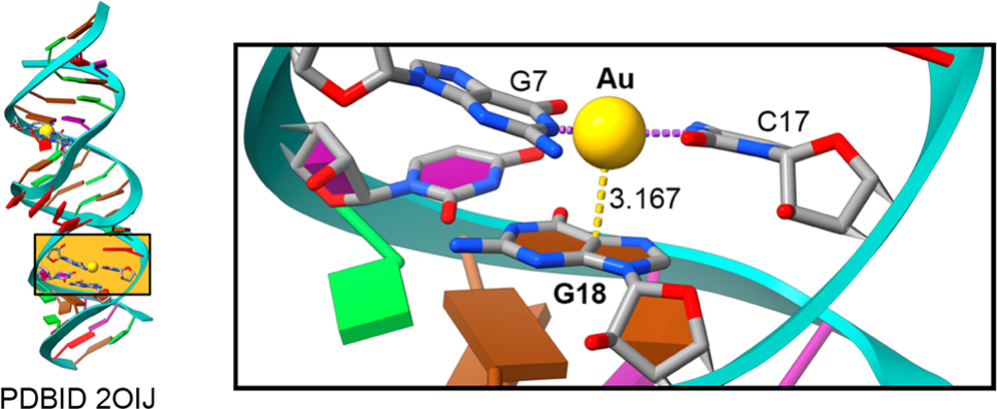
Regium−π interactions in the crystal structure 2OIJ. The interactions are magnified inside the rectangular parts of the figure. The yellow dashed line indicates an Au···guanine Rg−π bond in Å. Purple dashed lines indicate coordination bonds.

The second example encompasses the work of Ennifar and co-workers (PDBID: 2OIJ),^64^ who carried out a systematic crystallographic study on the binding of several metal ions to RNA duplexes. One of the salts used by the authors was AuCl_3_, and once the X-ray structure was solved, they noticed that the Au^3+^ cation induced deprotonation of N1 in G7 and bound within the Watson-Crick faces of the two G7–C17 base pairs of the duplex (see Figure 3). The cation was coordinated following a (distorted) square-planar geometry, in agreement with the usual behavior of cations like Pd(II), Pt(II), and Au(III) having a d8 electronic configuration.^65^ Interestingly, the Au(III) coordination complex is involved in a Rg−π bond with a G ring located just below (G18 in Figure 3), with a distance of 3.167 Å. The calculated interaction energy of this Rg−π bond was −33.8 kcal/mol, larger than that for 5CCW structure. A likely explanation would be related to the establishment of a HB interaction between the amino group from G7 and the N5 belonging to G18 (not shown in Figure 3). Since Au^3+^ is considered a chemical probe for the recognition of G-C base pairs, the formation of Rg−π bonds might also be considered as an additional tool with applications in nucleic acid probing.

**Figure 4:**
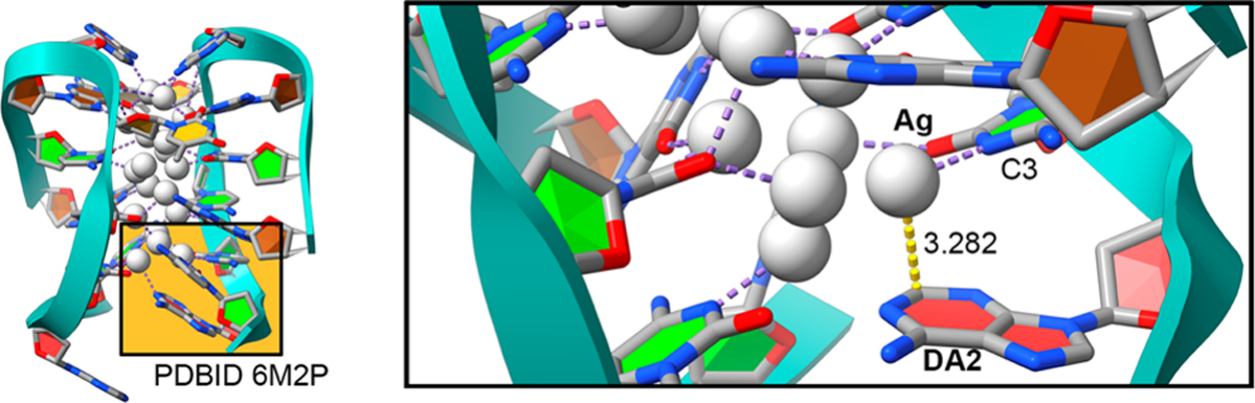
Regium−π interactions in the crystal structure 6M2P. The interactions are magnified inside the rectangular parts of the figure. The yellow dashed line indicates an Au···adenine Rg−π bond in Å. Purple dashed lines indicate coordination bonds.

The last example involves the study from Cerretani and collaborators (PDBID: 6M2P),^66^ who carried out a photophysical study on DNA-A_10_:Ag_16_ nanoclusters (NCs) in solution. DNA-stabilized silver nanoclusters (DNA:AgNCs) are a class of fluorophores that contain a limited number of silver atoms (usually less than 30) wrapped in one or several single-stranded DNA oligomers.^67^ These emitters have been used for several applications which vary from sensing to fluorescence imaging.^68^ In Figure 4, a general view of the Ag nanocluster complexed to a DNA oligomer is shown, and interestingly, one of the Ag atoms located at the bottom side of the Ag NC is interacting through a noncovalent bond with the π-system of A2 (*d*_Ag···A_ = 3.282 Å), thus establishing a Rg−π interaction. The computed interaction energy for this complex resulted in −12.4 kcal/mol, a moderately strong value.

The rest of the interaction energy values involving other Rg−π bonds obtained from the search are given in Table 1, along with their respective distances and interaction angles. In general, the results obtained lie within the same range as the ones reported for protein-involved Rg−π bonds.^47^ Also, the angle of interaction is close to 90°, thus pointing out to a certain directionality of the interaction, similar to that observed for cation and anion−π interactions.^69^

### Electrostatic Potential Surface Analysis

**Figure 5:**
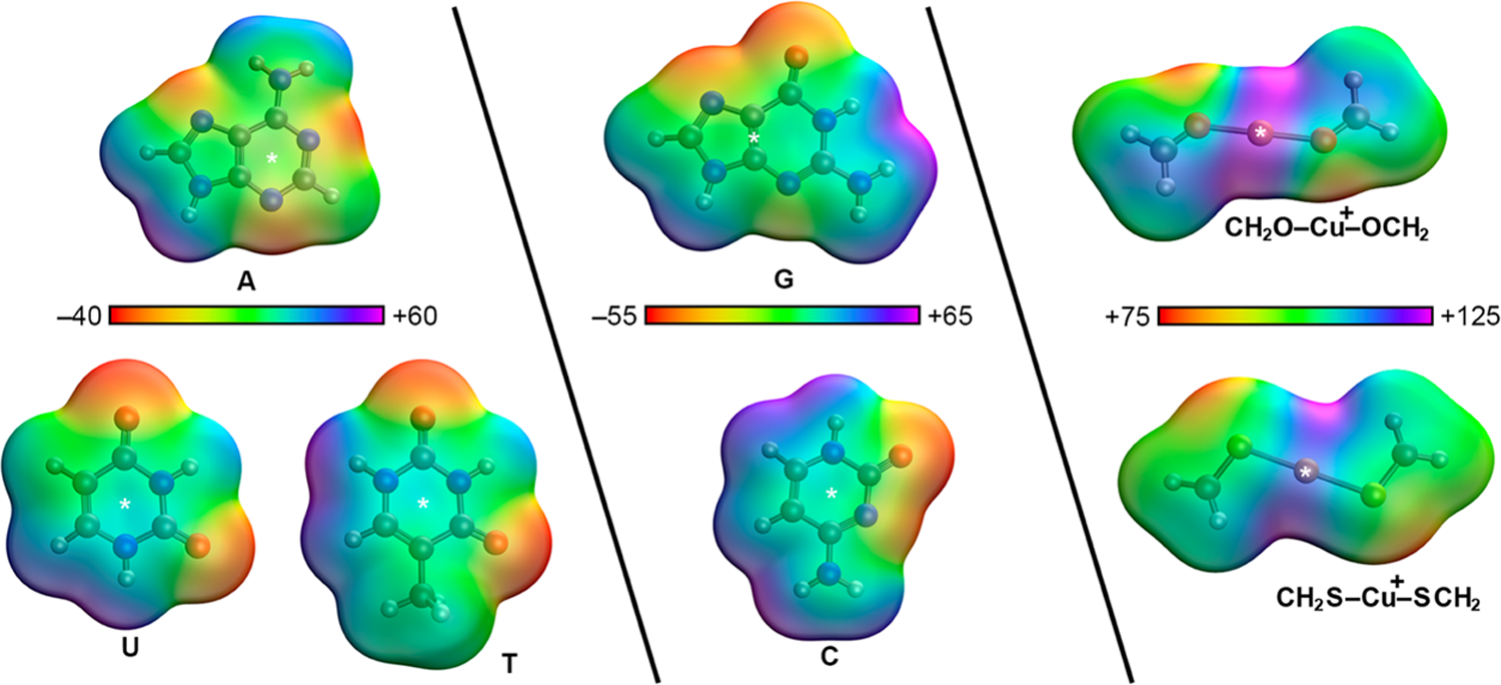
Molecular electrostatic potential (MEP) values of A, G, C, T, and U nucleobases and [Cu(CH_2_O)_2_]^+^ and [Cu(CH_2_S)_2_]^+^ moieties. Energy values at selected points of the surface (denoted by an asterisk) are given in kcal/mol in (Table 2) using an isovalue of 0.001 au. The rest of the MEP values are also gathered in Table 2.

With the purpose of studying in detail the nature of the Rg−π bonds present in those systems, we have complemented the findings gathered from the PDB survey with Quantum Mechanics (QM) calculations. We started by inspecting the molecular electrostatic potential (MEP) surfaces of A, G, C, T, and U bases as well as [RgL_2_]^+^ (Rg = Cu(I), Ag(I), and Au(I) and L = CH_2_O, CH_2_S) coordination complexes, and the results are gathered in Figure 5 and Table 2. As noted, for A, G, and C bases, a negative MEP value was found over the ring portion, the middle C–C double bond (in G, −6.9 kcal/mol) being the most negative region followed by the six-membered ring of A (−4.5 kcal/mol) and C (−3.4 kcal/mol) nucleobases apart from the MEP values at the O atoms in the molecular plane. On the contrary, T and U exhibited positive MEP values (+12.6 and +13.8 kcal/mol, respectively). Hence, Rg−π complexes involving A, G, and C are expected to be more favorable from an electrostatic point of view than those involving T and U.

**Table 2:** Molecular Electrostatic Potential (MEP) Surfaces of A, G, C, T, and U Nucleobases and [RgL_2_]^+^ (Rg = Cu, Ag, and Au and L = CH_2_O and CH_2_S) Coordination Complexes^a^

nucleobase/compound	*V*_ESP_
A	–4.5
G	–6.9
C	–3.4
T	+12.6
U	+13.8
[O–Cu–O]^+^	+112.9
[O–Ag–O]^+^	+113.6
[O–Au–O]^+^	+94.8
[S–Cu–S]^+^	+106.0
[S–Ag–S]^+^	+101.6
[S–Au–S]^+^	+91.0

a The energies gathered at selected points in the surface are given in kcal/mol (VESP) using an isovalue of 0.001 au.

Figure 5 also includes the electrostatic potential of two [RgL_2_]^+^ moieties ([Cu(CH_2_O)_2_]^+^ and [Cu(CH_2_S)_2_]^+^ were used as selected examples, while the rest of MEP values are included in Table 2). Owing to the cationic nature of these coordination complexes, very positive MEP values were found in all of the cases, [Ag(CH_2_O)_2_]^+^ and [Cu(CH_2_S)_2_]^+^ moieties being the ones exhibiting the most positive potential values (+113.6 and +106.0 kcal/mol, respectively). Interestingly, in both cases, the Au coordination complexes achieved the less positive MEP values of the series (+94.8 and +91.0 kcal/mol, respectively), contrary to the well-established trend of σ- and π-hole interactions from the p-block of elements.^21^ Hence, complexes involving these compounds are expected to show more favorable interaction energy values than the rest of the compounds used. As a concluding remark, it is also important to mention the study carried out by Alkorta and collaborators,^70^ who reported the inclusion of a molecular polarization potential (MPP) correction on the MEP values of nucleobases to account for polarization effects due to an external positive/negative charge. In this regard, while the MEP minima and maxima would be affected by including the MPP correction, the general behavior observed would remain the same. Therefore, the computed MEP values are sufficient to understand the electrostatics of the compounds shown in Figure 5.

### Energetics Study

**Figure 6:**
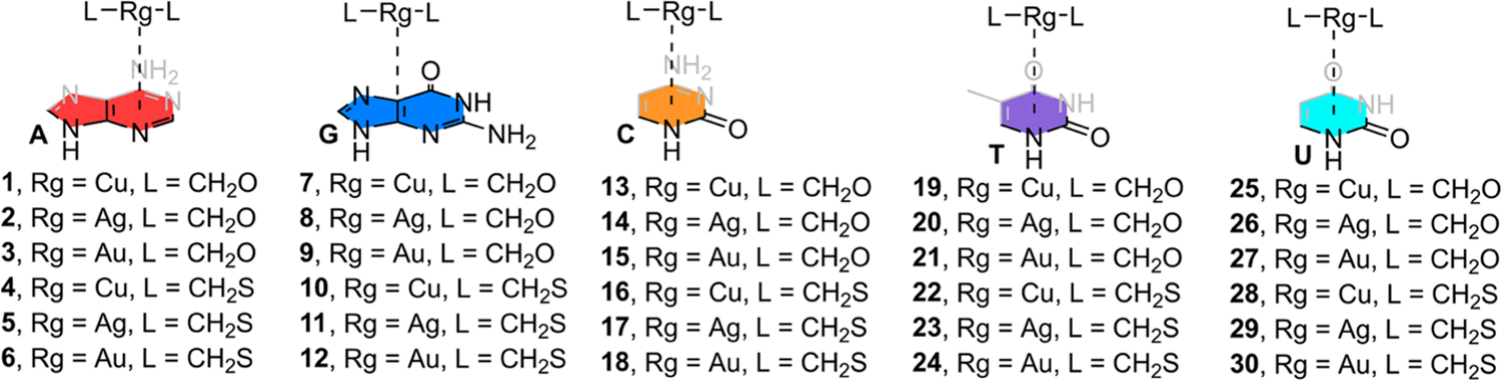
Complexes **1**–**30** used in this study.

**Figure 7:**
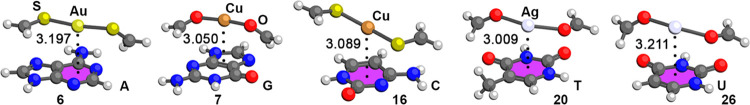
Optimized geometries of complexes **6**, **7**, **16**, **20**, and **26** at the RI-MP2/def2-TZVP//PBE0-D3/def2-SVP level of theory.

With the purpose of studying the ability of the nucleobases to undergo Rg−π binding, we performed a series of calculations on several [L_2_Rg]^+^···π complexes (see Figures 6 and 7 and Table 3). First, in all of the cases, negative and moderately strong binding energy values were obtained, ranging between −23.1 and −6.0 kcal/mol. Second, complexes involving G (**7**–**12**) obtained the most favorable interaction energy values of the study, in agreement with the MEP analysis discussed above. Lastly, complexes involving A and C (**1**–**6** and **13**–**18**, respectively) presented larger interaction energy values compared to those involving T and U (**19**–**24** and **25**–**30**, respectively), also in line with the results derived from the MEP analysis.

**Table 3:** Regium Bond Donor (RgB Donor) and Acceptor (RgB Acceptor) Counterparts, BSSE-Corrected Interaction Energies (in kcal·mol^–1^), and Equilibrium Distances (*d*, in Å) of Complexes **1**–**30**^a^

complex	RgB donor	RgB acceptor	Δ*E*	*d*
**1**	[O–Cu–O]^+^	A	–12.0	2.893
**2**	[O–Ag–O]^+^	A	–15.1	2.993
**3**	[O–Au–O]^+^	A	–14.0	3.094
**4**	[S–Cu–S]^+^	A	–15.1	3.094
**5**	[S–Ag–S]^+^	A	–15.5	3.194
**6**	[S–Au–S]^+^	A	–16.0	3.197
**7**	[O–Cu–O]^+^	G	–20.1	3.050
**8**	[O–Ag–O]^+^	G	–18.5	3.050
**9**	[O–Au–O]^+^	G	–23.1	3.150
**10**	[S–Cu–S]^+^	G	–16.1	3.050
**11**	[S–Ag–S]^+^	G	–16.8	3.150
**12**	[S–Au–S]^+^	G	–17.9	3.150
**13**	[O–Cu–O]^+^	C	–14.7	2.994
**14**	[O–Ag–O]^+^	C	–9.3	3.089
**15**	[O–Au–O]^+^	C	–15.6	3.201
**16**	[S–Cu–S]^+^	C	–10.5	3.089
**17**	[S–Ag–S]^+^	C	–11.3	3.090
**18**	[S–Au–S]^+^	C	–12.5	3.191
**19**	[O–Cu–O]^+^	T	–6.0	2.916
**20**	[O–Ag–O]^+^	T	–7.4	3.009
**21**	[O–Au–O]^+^	T	–8.3	3.115
**22**	[S–Cu–S]^+^	T	–6.7	3.006
**23**	[S–Ag–S]^+^	T	–8.4	3.406
**24**	[S–Au–S]^+^	T	–10.8	3.770
**25**	[O–Cu–O]^+^	U	–8.9	3.265
**26**	[O–Ag–O]^+^	U	–8.8	3.261
**27**	[O–Au–O]^+^	U	–11.8	3.474
**28**	[S–Cu–S]^+^	U	–7.6	3.202
**29**	[S–Ag–S]^+^	U	–8.8	3.306
**30**	[S–Au–S]^+^	U	–7.9	3.309

a Values given as the distance between the Rg atom and the centroid of the six-membered ring in A, C, T, and U. In the case of G, the centroid was placed over the central C–C double bond.

Complexes **1**–**6** involved adenine as a nucleobase, with a stability between −16.0 and −12.0 kcal/mol. Concretely, complex **6** (involving [Au(CH_2_S)_2_]^+^) obtained the most favorable binding energy value (−16.0 kcal/mol), while complex **1** (involving [Cu(CH_2_O)_2_]^+^) achieved the lowest stability of the set (−12.0 kcal/mol). The results obtained were similar among the Rg atoms studied when using the CH_2_S ligand (−15.1 for complex **4**, −15.5 for complex **5**, and −16.0 kcal/mol for complex **6**), showing a slight reinforcement of the interaction strength. On the other hand, more noticeable differences were observed in the case of complexes **1**–**3** (−12.0, −15.1, and −14.0 kcal/mol, respectively), which partially agree with the results from the MEP analysis.

Complexes **7**–**12** involved guanine as the nucleobase, with stability ranging between −23.1 and −16.1 kcal/mol. In this set of complexes, complex **9** achieved the most favorable interaction energy value (−23.1 kcal/mol), while complex **10** obtained the lowest interaction energy value (−16.1 kcal/mol). Also, the strength of the interaction was reinforced on going from lighter to heavier Regium atoms (using CH_2_S as a ligand), similarly to that observed in the case of complexes involving A.

Complexes **13**–**18** involved cytosine as a base, with a stability that varies from −15.6 to −9.3 kcal/mol. In this set of complexes, complex **15** [involving [Au(CH_2_O)_2_]^+^ obtained the most favorable interaction energy value (−15.6 kcal/mol), while complex **14** achieved the lowest interaction energy value of the study (−9.3 kcal/mol)]. Furthermore, a decrease in stability was observed on going from Cu to Ag while an increase in strength was observed from Ag to Au involving complexes (**13**–**15**), similar to that obtained for guanine complexes **7**–**9**. This was not observed in the case of using CH_2_S as a metal ligand, where an increase in the interaction strength was obtained, in line with the results obtained for G and A involving complexes (**4**–**6** and **10**–**12**, respectively).

Complexes **19**–**24** involved thymine as the nucleobase, with energetic values ranging from −10.8 to −6.0 kcal/mol. As observed, complex **24** involving [Au(CH_2_S)_2_]^+^ obtained the most favorable binding energy value (−10.8 kcal/mol), while complex **19** involving [Cu(CH_2_O)_2_]^+^ resulted in the poorest binding energy value (−6.0 kcal/mol) of the set. In this case, the same trend was followed by both the O- and S-coordinated complexes, that is, a strengthening of the interaction from using Cu to Au, contrary to the MEP values shown above.

Finally, complexes **25**–**30** involved uracil as nucleobase, with stability varying between −11.8 and −7.6 kcal/mol. As noted, complex **27** involving [Au(CH_2_O)_2_]^+^ yielded the most favorable interaction energy value of the set, while complex **28** involving [Cu(CH_2_S)_2_]^+^ achieved the lowest binding energy value. In this case, similar interaction energy values were obtained when comparing O–Cu–O and O–Ag–O involving complexes (**25** and **26**), while an increase in the interaction strength was obtained when using O–Au–O moiety (complex **27**, −11.8 kcal/mol). On the other hand, in those complexes involving CH_2_S as a metal ligand (**28**–**30**), an increase in the interaction strength was observed from Cu to Ag and a decrease while going from Ag to Au.

These results do not completely agree with the MEP trends discussed above for the metal complexes, and thus, the MEP values obtained for the RgL_2_ metal complexes cannot strictly predict the stability of these Rg−π complexes. This points out to the importance of other energy contributions (e.g., polarization or dispersion) as well as to additional interactions as a stability source of these complexes (see QTAIM analysis below).

### AIM and NCIplot Analyses

**Figure 8:**
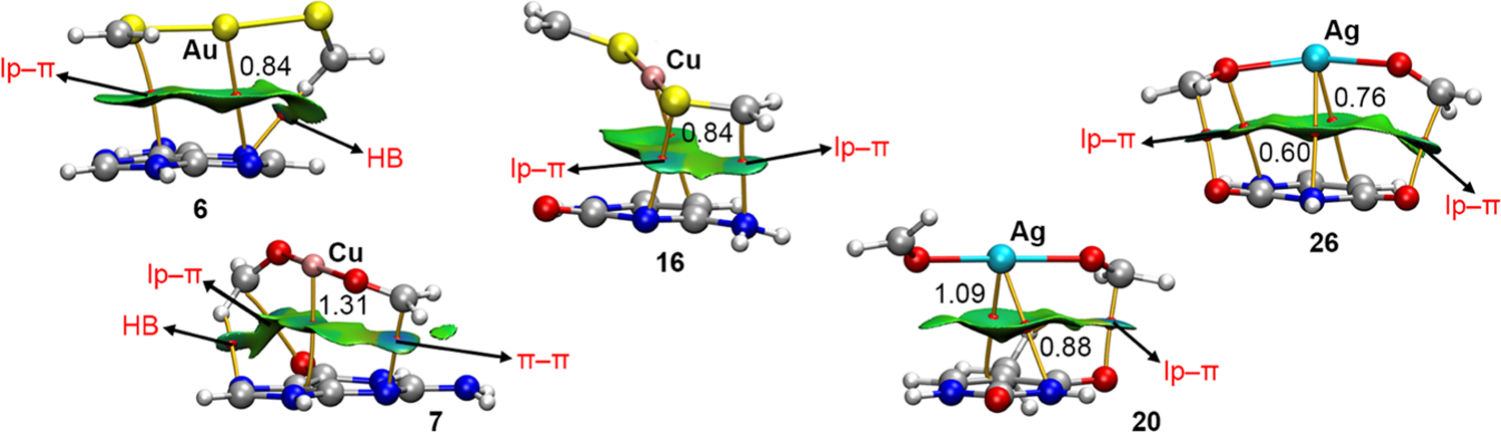
NCIplot analysis and AIM distribution of intermolecular bond critical points (BCP in red spheres) and bond paths in complexes **6**, **7**, **16**, **20**, and **26**. The value of density at the BCPs characterizing the Rg−π interaction is also indicated. Ancillary interactions are highlighted in red. NCIplot surfaces involving only intermolecular contacts between the Rg coordination complex and the nucleobases are also indicated. NCIplot color range −0.035 au ≤ (signλ_2_)ρ ≤ +0.035 au. Isosurface value |RGD| = 0.5 and ρ cutoff 0.04 au.

The QTAIM analyses^71^ of the Rg−π bonds in complexes **6**, **7**, **16**, **20**, and **26** are shown in Figure 8. As noted, in all of the cases, one (in complexes **6**, **7**, and **16**) or two (in complexes **20** and **26**) bond critical points (BCPs) and bond paths connect the Rg atom to the N/C atoms from the nucleobase π-system. In addition, several additional interactions [lone pair−π (lp−π), π–π stacking, and hydrogen bonding (HB)] were also observed, thus helping in the rationalization of the interaction energies discussed above.

For instance, in the case of complex **6** involving [Au(CH_2_S)_2_]^+^ and adenine, two additional BCPs and bond paths connected (i) the π-system of A with the lone pairs of an S atom and (ii) an N atom of the adenine moiety to a CH bond from the [Au(CH_2_S)_2_]^+^ molecule, thus characterizing lp−π and HB interactions. In the case of complex **7** involving guanine, ancillary HB and π–π stacking interactions were denoted by the BCPs connecting a CH group and the C–O π-system of the organic ligand to an N and a C–N π bond from the G ring.

In complex **16**, two lp−π interactions were characterized by two BCPs and bond paths connecting the lone pairs of an S atom from the organic ligand and an N belonging to an amino group from cytosine to a C–N π bond from cytosine and the C–S π-system of the ligand, respectively. In the case of complexes **20** and **26**, ancillary lp−π interactions were also present, as observed from the BCPs connecting (i) the sp^2^ O lone pairs (from both T and U bases) to the C–O π-system of the organic ligand and (ii) from the O lone pairs of the ligand to a C–N π-bond of the nucleobase in complex **26**.

Lastly, for all five selected examples, the noncovalent interactions plot (NCIplot) analysis was also carried out. Interestingly, an extended greenish isosurface was observed in all cases, denoting a noncovalent contact between the [L_2_Rg]^+^ and the nucleobase counterparts. In addition, in complexes **7** and **16**, a bluish isosurface was obtained involving a π–π stacking and an lp−π interaction, respectively, thus indicating a stronger contribution to binding upon the formation of the Rg−π complex. In the other cases, the isosurface color for all of the interactions present was similar, thus likely pointing out to an equal contribution of all NCIs.

In Table 4, the values of the density at the BCP that characterizes the Rg−π bond as well as the ancillary interactions (px100, in au) present in complexes **6**, **7**, **16**, **20**, and **26** are shown. As noted, the BCP density values involving the Rg−π bond are of lower magnitude than those involving the lp−π, π–π, and HB interactions present in those complexes. More in detail, in complex **6**, both the Rg−π and lp−π interaction achieved similar BCP density values (0.84 and 1.05 au, respectively), thus expecting a similar strength, while the HB between the CH_2_ group from the organic ligand and an N atom from adenine exhibited a larger BCP density value (1.46 au). In **7**, the Rg−π interaction presented a BCP density higher than the CH···N HB present in this complex (1.31 and 1.27 au, respectively). On the other hand, the BCP attributed to a π–π stacking interaction obtained the largest density value (2.10 au) due to the parallel overlap between the π-systems of the organic ligand and the guanine six-membered ring. Finally, the BCP density attributed to the lp−π interaction resulted in an intermediate value between those (1.72 au). In complex **16**, the sum of the lp−π BCP density values was larger than the Rg−π BCP density (2.95 and 0.84 au, respectively), thus remarking the stabilizing role of the former in the stabilization of this Rg−π complex. Finally, in complex **20**, the strength of both the Rg−π and lp−π interactions is similar (1.97 and 2.14 au, respectively), while in the case of complex **26**, the contribution of the lp−π interactions was remarkable (3.82 au) compared to the Rg−π bond (1.36 au). Despite this, the individual contributions of each lp−π interaction (1.63, 1.40, and 0.79 au) are within the same range or even lower than the Rg−π bond.

**Table 4:** Values of the Density at the Bond Critical Points (ρx100, in au) That Characterize the Rg−π and the Ancillary Interactions Present in Complexes **6**, **7**, **16**, **20**, and **26**^a^

		Rg−π				
complex	ρx100	∇^2^ρx100		Vx100	Gx100	Hx100
**6**	0.84 (Rg−π)	2.94		–0.51	0.62	0.11
	1.05 (lp−π)					
	1.46 (HB)					
**7**	1.31 (Rg−π)	2.72		–0.84	0.76	–0.08
	1.27 (HB)					
	2.10 (π–π)					
	1.72 (lp−π)					
**16**	0.84 (Rg−π)	1.83		–0.49	0.48	–0.02
	2.95 (lp−π)					
**20**	1.97 (Rg−π)	(Ag···C)	2.98	–0.63	0.69	0.06
	2.14 (lp−π)	(Ag···N)	3.12	–0.55	0.67	0.11
**26**	1.36 (Rg−π)	(Ag···N)	2.09	–0.32	0.42	0.10
	3.82 (lp−π)	(Ag···C)	2.16	–0.39	0.47	0.07

a In addition, the values of the laplacian of ρ (∇2ρx100, in au), the potential (Vx100, in au) and kinetic (Gx100, in au) energy densities as well as the total energy density (Hx100, in au) regarding the Rg−π interaction are also indicated.

**Figure 9:**
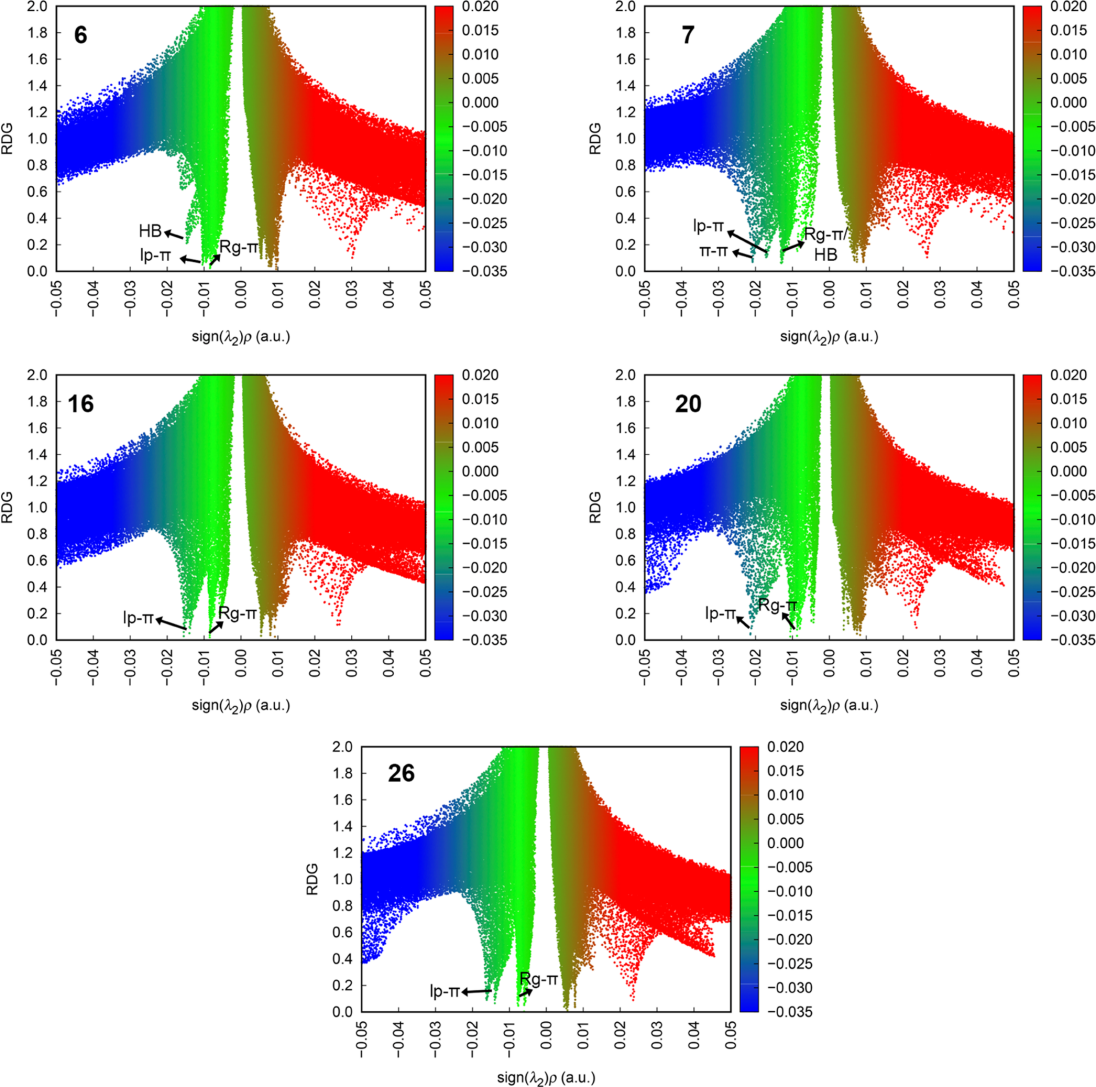
Graphic representation of the reduced density gradient (RDG) vs the sign(λ_2_)ρ with an indication of the Rg−π as well as the ancillary interactions present in complexes **6**, **7**, **16**, **20**, and **26**.

In addition, we also indicated the values of the laplacian at the BCP that characterizes the Rg−π bond (∇^2^ρx100), resulting in positive values in all cases, as it is common in closed shell calculations. Furthermore, the values of the potential (Vx100) and kinetic (Gx100) energy densities lie within the same range in all of the cases, confirming the noncovalent nature of the Rg−π interaction (|Vr|/Gr) ≈ 1. Finally, in Figure 9, the plots of the reduced density gradient (RDG) vs the sign(λ_2_)ρ, indicating both the Rg−π as well as the additional interactions present in complexes **6**, **7**, **16**, **20**, and **26**, are shown, being consistent with the data gathered in Table 4.

### NBO Analysis

To further investigate the participation of orbital contributions in the stabilization of the noncovalent complexes studied, we carried out NBO calculations focusing on the second-order perturbation analysis,^72^ which is useful to evaluate donor–acceptor interactions (see Table 5).

**Table 5:** Donor and Acceptor NBOs with Indication of the Second-Order Interaction Energy *E*^(2)^ in Complexes **6**, **7**, **16**, **20**, and **26**. LP, BD, BD*, and LV Stand for Lone Pair, Bonding Orbital, Antibonding Orbital, and Lone Valence, Respectively^a^

complex	type of interaction	donor	acceptor	*E*^(2)^
**6**	Rg−π	BD C–N	BD* Au–S	0.47
	Rg−π	LP Au	BD* C–N	0.56
	lp−π	LP S	BD* C–N	0.20
	HB	LP N	BD* C–H	2.17
**7**	Rg−π	BD C–C	LV Cu	1.38
	Rg−π	LP Cu	BD* C–C	0.08
	HB	LP N	BD* C–H	1.39
	π–π	BD C–N	BD* C–O	4.34
	lp−π	LP O	BD* C–O	2.38
**16**	Rg−π	BD C–C	LV Cu	1.64
	lp−π	LP S	BD* C–N	0.20
	lp−π	LP N	BD* C–S	6.13
**20**	Rg−π	BD C–C	LV Ag	1.34
	Rg−π	LP Ag	BD* C–C	0.14
	Rg−π	LP Ag	BD* C–O	0.41
	lp−π	LP O	BD* C–O	2.53
**26**	Rg−π	BD C–C	LV Ag	1.02
	Rg−π	LP Ag	BD* C–C	0.06
	Rg−π	LP Ag	BD* C–O	0.17
	lp−π	LP O	BD* C–O	0.94

a Energy values are in kcal/mol.

First, in the case of complex **6**, two main orbital contributions of equal magnitude were found: (i) the donation from a π bonding (BD) C–N orbital of adenine to a σ antibonding (BD*) Au–S orbital of the metal complex (0.47 kcal/mol) and (ii) the back-donation from a lone pair (LP) of the Au atom to a π antibonding (BD*) C–N orbital of adenine (0.56 kcal/mol). Second, for complexes **7**, **16**, **20**, and **26**, this analysis reveals an orbital contribution involving the electron donation from bonding (BD) C–C and C–N π orbitals to an empty orbital (LV) of the metal atom (basically composed of an s atomic orbital). In addition, in complexes **7**, **20**, and **26**, we have also found a back-donation effect from a lone pair (LP) of the metal atom to a BD* C–N, C–C, or C–O orbital from the ring moiety, although this orbital contribution was of much smaller magnitude than the former. These results confirm the π-hole nature of the interaction and are consistent with recent reports by some of us regarding π-hole regium bonds.^47,73^

We have also included the orbital contributions involving the ancillary lone pair−π (lp−π), hydrogen bond (HB), and π–π stacking interactions highlighted in the AIM analysis. As can be seen in Table 5, these encompass (i) lp−π interactions involving the donation from a LP belonging to a S, N, or O atoms to a BD* C–N, C–S, or C–O orbital (complexes **6**, **7**, **16**, **20**, and **26**), (ii) π–π stacking interactions involving the donation from a π BD C–N orbital to a π BD* C–O orbital (complex **7**), and (iii) HB interactions involving the donation from a LP of an N atom to a BD* C–H orbital (complexes **6** and **7**). Regarding their magnitude, it spans from modest (LP S → BD* C–N, 0.20 kcal/mol in complex **6**) to moderately strong (BD C–N → BD* C–O, 4.34 kcal/mol and LP N → BD* C–S, 6.13 kcal/mol in complexes **7** and **16**, respectively), in line with the results obtained from the NCIplot analysis discussed above. Hence, the establishment of these additional NCIs is important for stabilizing the Rg−π complexes studied herein while also acting as anchorage points to prevent the coordination of the nucleobase N/O atoms to the metal complex, since N/O atoms usually participate in HB interactions in the real systems.

## Conclusions

In conclusion, we have conducted a PDB inspection looking for Regium−π interactions involving Cu(I/II)/Ag(I)/Au(I/III)-coordinated complexes and nucleobases (A, G, C, T, and U), resulting in a total number of 11 X-ray structures. The stability and directionality of the interaction in the selected examples were evaluated at the RI-MP2/def2-TZVP level of theory. In addition, a computational study was carried out to investigate the stability of a series of [L_2_Rg]^+^···π bonds (L = CH_2_O and CH_2_S, Rg = Cu(I), Ag(I), and Au(I)) at the RI-MP2/def2-TZVP//PBE0-D3/def2-SVP level of theory. The Rg−π interactions studied were characterized through QTAIM and NCIplot analyses. Finally, the NBO analyses shed light into donor–acceptor orbital interactions, which played a noticeable role in the stability of the noncovalent complexes studied herein. We hope the findings gathered in this work will be useful for the community working on DNA/RNA engineering and chemical biology as well as to make more visible the Regium bonding interaction among the biological community.

## Supporting Information Available

Cartesian coordinates of complexes **1**–**30** and additional QTAIM and NCIplot analyses (PDF)
