# Supplementary material for: Regium−π Bonds Involving Nucleobases: Theoretical Study and Biological Implications
Source: Inorg Chem. 2023 Apr 21;62(17):6740–50. doi: 10.1021/acs.inorgchem.3c00369 (PMC10155183; doi:10.1021/acs.inorgchem.3c00369)
Supplement: Supplementary file 1 — ic3c00369_si_001.pdf [file ic3c00369_si_001.pdf]

Regium-π bonds involving nucleobases: Theoretical study and biological implications.

Sergi Burguera[a], Antonio Frontera[a] and Antonio Bauzá*[,a]

[a]Department of Chemistry, Universitat de les Illes Balears, Crta. de Valldemossa km 7.5, 07122 Palma (Islas Baleares), SPAIN.
Fax: (+) 34 971 173426; E-mail: antonio.bauza@uib.es

Electronic Supplementary Information

Index

Cartesian coordinates of complexes 1 to 30 page 2
Additional QTAIM and NCIplot analyses page 19
Cartesian coordinates of the selected PDB structures page 20

Cartesian coordinates of complexes 1 to 30

1.

N	2.1581815	-0.0581423	-1.7260034
C	2.9881626	0.3925600	-0.7383154
N	2.5869485	0.0626892	0.4620703
C	1.4430393	-0.6646887	0.2479330
C	0.5509621	-1.3234356	1.1178392
N	0.7076244	-1.3312678	2.4436557
N	-0.5110641	-1.9395070	0.5755382
C	-0.6381711	-1.9591132	-0.7542007
N	0.1269972	-1.3884682	-1.6714412
C	1.1502975	-0.7486478	-1.1114934
H	2.2976118	0.0194060	-2.7210306
H	3.8846675	0.9543254	-0.9601897
H	0.1088319	-1.9157158	3.0013709
H	1.5516269	-0.9657879	2.8489441
H	-1.4893787	-2.5197428	-1.1328483
C	-3.5154341	-0.6458693	-0.1647710
H	-4.5244586	-0.8399552	-0.5498642
C	1.2187254	2.5769912	0.4458606
H	1.9004477	2.8942515	1.2425312
H	1.4988041	2.7778755	-0.5959636
H	-3.0708885	-1.3408724	0.5607446
Cu	-1.2372107	1.0712887	-0.0660653
O	0.1639083	2.0461158	0.7344668
O	-2.9123681	0.3326366	-0.5606569

2.

N	0.1124013	-1.8879493	1.9200660
C	-0.5138715	-2.8244693	1.1479341
N	-0.2195898	-2.7258638	-0.1203188
C	0.6641020	-1.6744154	-0.1804226
C	1.3505476	-1.0707481	-1.2461285
N	1.2306694	-1.4960178	-2.5198086
N	2.1186676	-0.0093188	-0.9842843
C	2.2347322	0.4064765	0.2760637
N	1.6503282	-0.0777168	1.3672213
C	0.8785089	-1.1263336	1.0822036
H	0.0646349	-1.8040257	2.9228596
H	-1.1851531	-3.5551905	1.5755578
H	1.8786555	-1.1097464	-3.1882932
H	0.9237940	-2.4424153	-2.6751683
H	2.8964217	1.2560544	0.4219003
C	0.7945365	3.1769121	1.4710306

H	1.1247269	4.1924728	1.7317332
C	-2.1906394	-1.3375869	-1.5244970
H	-1.4080353	-1.4621371	-2.2827230
H	-2.9520602	-2.1231184	-1.4325844
H	1.1156275	2.3346901	2.1019948
Ag	-0.8503811	1.2387811	-0.3539156
O	-2.2559062	-0.3527129	-0.8176332
O	0.0961572	3.0141010	0.4936525

3.

N	-0.1995877	-2.4519507	1.8838984
C	-1.1160726	-3.0545223	1.0785712
N	-0.8694825	-2.8859317	-0.1979528
C	0.2938965	-2.1521250	-0.2241642
C	1.0878163	-1.6583471	-1.2768328
N	0.8264938	-1.9080829	-2.5639302
N	2.1285755	-0.8783081	-0.9595128
C	2.3974978	-0.6576004	0.3262189
N	1.7644273	-1.1021653	1.4030184
C	0.7161694	-1.8449000	1.0648680
H	-0.1688783	-2.4729932	2.8908610
H	-1.9556795	-3.6042979	1.4798971
H	1.4890152	-1.6037904	-3.2568188
H	0.1465690	-2.6044582	-2.8123423
H	3.2802473	-0.0503701	0.5157523
C	1.9721573	2.6197530	0.7647580
H	2.5116318	3.2825790	1.4506216
C	-2.4697862	-0.8696693	-0.7860581
H	-2.9568502	-1.4965675	-1.5375610
H	-2.7704489	-0.9662534	0.2599592
H	2.4772963	2.2405772	-0.1307242
Au	-0.4188303	1.1208415	-0.0358562
O	-1.6588784	-0.0333681	-1.1623205
O	0.8184007	2.3337420	1.0319214

4.

N	2.3687789	-0.2909892	-1.5891389
C	3.2445368	-0.0048359	-0.5689784
N	2.7992061	-0.3562990	0.6017652
C	1.5753794	-0.9238818	0.3415113
C	0.6253070	-1.5619710	1.1630839
N	0.7620073	-1.6366884	2.4921729
N	-0.4579306	-2.0898941	0.5778536
C	-0.5736167	-2.0294568	-0.7544735
N	0.2304248	-1.4416610	-1.6254791
C	1.2916508	-0.9085359	-1.0228737
H	2.5454065	-0.2133937	-2.5782147

H	4.2018651	0.4596242	-0.7581547
H	0.1506395	-2.2466706	3.0076012
H	1.6341828	-1.3607629	2.9106441
H	-1.4540399	-2.5125228	-1.1692077
C	-3.4248447	-0.8735040	0.6021997
H	-4.4272907	-1.2944115	0.6386436
C	1.4411710	2.7398800	-0.7282315
H	2.3862273	3.2736288	-0.6626326
H	1.0111947	2.6001900	-1.7145222
H	-2.6014867	-1.4478772	1.0267402
Cu	-1.1050284	1.1618734	0.1118649
S	0.7529859	2.2152835	0.6283740
S	-3.2217938	0.5573053	-0.1108326

5.

N	0.3839613	-2.5430711	1.5996246
C	0.0153932	-3.4046450	0.5957985
N	0.3356230	-2.9766702	-0.5906336
C	0.9725549	-1.7814494	-0.3582855
C	1.6244951	-0.8677269	-1.2084453
N	1.6409694	-1.0130262	-2.5396357
N	2.2250106	0.1916681	-0.6503832
C	2.2191938	0.3156740	0.6824427
N	1.6316296	-0.4584946	1.5798759
C	1.0256476	-1.4948671	1.0037551
H	0.3396873	-2.7140673	2.5915703
H	-0.4855758	-4.3387264	0.8068671
H	2.2638312	-0.4374207	-3.0801346
H	1.3132305	-1.8756755	-2.9400472
H	2.7573796	1.1746639	1.0739612
C	1.2845353	3.2713601	-0.6540332
H	1.8217566	4.2155583	-0.7190133
C	-2.7962954	-1.5989171	0.8480408
H	-3.3639473	-2.5266501	0.8206801
H	-2.5884311	-1.1711492	1.8236098
H	1.7447581	2.3824078	-1.0864443
Ag	-1.0623265	1.0393384	-0.0696968
S	-2.3242073	-0.9395033	-0.5390289
S	-0.1325536	3.2513573	0.1090646

6.

N	0.0315730	-2.7579961	1.6316283
C	-0.4849276	-3.5652613	0.6463200
N	-0.0826083	-3.2372127	-0.5470121
C	0.7616684	-2.1731955	-0.3388064
C	1.5668921	-1.4080981	-1.2058214
N	1.5443451	-1.5728642	-2.5339246

N	2.3648043	-0.4787704	-0.6666155
C	2.3852673	-0.3339522	0.6632897
N	1.6594124	-0.9585379	1.5745635
C	0.8626310	-1.8669206	1.0170597
H	-0.0488959	-2.8909727	2.6272590
H	-1.1486473	-4.3863772	0.8771126
H	2.2536644	-1.1251926	-3.0886957
H	1.0554623	-2.3619817	-2.9211411
H	3.0879562	0.4044064	1.0409648
C	1.9959675	2.7089473	-0.5320511
H	2.7004834	3.5372464	-0.5025727
C	-2.7958934	-1.2761573	0.7180938
H	-3.4907306	-2.1071459	0.6261860
H	-2.6114408	-0.8703853	1.7063080
H	2.2761314	1.7955131	-1.0559450
Au	-0.6901484	1.0097780	-0.1005391
S	-2.1135680	-0.7130634	-0.6239328
S	0.5971465	2.8912782	0.2440566

7.

N	-0.2742211	-0.5807016	2.2670424
C	-1.4663901	-1.2423346	2.1085824
N	-1.6465311	-1.6630426	0.8912654
C	-0.5093781	-1.2865086	0.2209434
C	1.9043109	-0.2224956	-0.4832896
N	1.5439539	-0.0347416	0.7680294
C	0.3476879	-0.5878256	1.0589814
C	-0.1875431	-1.3543016	-1.1684576
O	-0.8680191	-1.7173596	-2.1051906
N	3.0807329	0.2842734	-0.9081966
N	1.1117019	-0.8413936	-1.3994646
H	0.0918279	-0.1951726	3.1224564
H	-2.1551821	-1.3786806	2.9292364
H	3.7423849	0.5275204	-0.1894276
H	3.4713109	-0.0036736	-1.7889126
H	1.4054879	-0.9002636	-2.3653646
Cu	-1.4631256	1.6726113	-0.1221791
C	0.9626114	2.5274493	0.9838189
C	-2.9719566	-0.1957247	-1.6999681
O	-2.4906836	0.9121223	-1.5063511
O	-0.2461526	2.4048183	1.1166099
H	1.5867754	2.6957313	1.8681679
H	1.4449714	2.5547303	0.0006919
H	-3.3540316	-0.4350907	-2.6977921
H	-3.0605436	-0.9399457	-0.9012321

8.

N	-0.5332688	-1.2261825	2.0453375

C	-1.6123618	-1.8896545	1.5253325
N	-1.5655868	-1.9806955	0.2277455
C	-0.3885298	-1.3663215	-0.1255145
C	1.9700852	-0.0032315	-0.1005105
N	1.4337052	-0.2142985	1.0692655
C	0.2634292	-0.8753505	0.9969565
C	0.1553562	-1.0664865	-1.4165615
O	-0.3032808	-1.2654695	-2.5174205
N	3.1207932	0.7252485	-0.1783035
N	1.3945202	-0.3891865	-1.2736315
H	-0.3214798	-1.0818035	3.0200025
H	-2.4030358	-2.2734255	2.1529685
H	3.6179602	0.7837975	0.6977285
H	3.7173372	0.5927805	-0.9797725
H	1.8392982	-0.1709665	-2.1549275
Ag	-1.5631046	1.5193520	0.1524532
C	1.1067484	3.1759860	0.3351032
C	-3.2815356	-0.6499170	-1.1419098
O	-3.2550076	0.3458600	-0.4353428
O	0.1049454	2.7236100	0.8415002
H	1.8436424	3.6993640	0.9597732
H	1.3121904	3.0898480	-0.7410648
H	-4.1556506	-1.3117240	-1.0904748
H	-2.4971686	-0.8911330	-1.8687338

9.

N	0.0344375	0.8793244	-2.1854010
C	-0.7522055	1.9703094	-1.9118230
N	-0.7205715	2.3075364	-0.6569270
C	0.1476255	1.4161444	-0.0784090
C	1.7648835	-0.7202806	0.4360950
N	1.3941895	-0.5789626	-0.8183400
C	0.6021755	0.4961374	-1.0132720
C	0.3918205	1.1422304	1.2978230
O	-0.1223625	1.6270204	2.2890440
N	2.5597365	-1.7607206	0.7693780
N	1.3214565	0.0864904	1.4370480
H	0.1853385	0.4573414	-3.0871950
H	-1.3168815	2.4719344	-2.6837090
H	3.0717655	-2.1821866	0.0117930
H	3.0095805	-1.7863426	1.6686340
H	1.5621775	-0.1207446	2.3971810
Au	-2.0808658	-0.9094991	0.0953278
C	-0.1707528	-2.5658921	-1.3114442
C	-2.5358448	1.4028279	1.8289358
O	-2.7100018	0.1949699	1.6775918
O	-1.2291948	-1.9583381	-1.4290892

H	0.3322042	-2.9106411	-2.2201572
H	0.2465062	-2.8475421	-0.3413282
H	-2.7647428	1.8242799	2.8114378
H	-2.2204738	2.0646039	1.0168058

10.

N	-0.3729031	-1.3520292	2.0711525
C	-1.4273001	-2.0622022	1.5537935
N	-1.3271471	-2.2443292	0.2697135
C	-0.1411781	-1.6463682	-0.0773665
C	2.1736159	-0.1956902	-0.0578885
N	1.5973089	-0.3466892	1.1053055
C	0.4592819	-1.0687742	1.0327675
C	0.4580569	-1.4384792	-1.3654905
O	0.0835089	-1.7492272	-2.4669525
N	3.2837139	0.5876768	-0.1452265
N	1.6682909	-0.6964132	-1.2153785
H	-0.1987941	-1.1372032	3.0397315
H	-2.2321121	-2.4215402	2.1780975
H	3.7302929	0.7616578	0.7421045
H	3.9208599	0.4377408	-0.9108975
H	2.1480759	-0.5366232	-2.0905715
C	1.1936043	2.9807869	-0.3211285
C	-4.3484387	0.5772059	-0.6361555
S	-2.9061627	0.0429339	-1.1067845
S	0.2707403	2.4064979	0.8673385
Cu	-1.3567867	1.2337519	-0.0606095
H	2.0522043	3.5968579	-0.0690305
H	1.0075743	2.7896979	-1.3728315
H	-5.2381477	0.1139529	-1.0567345
H	-4.4981587	1.3668079	0.0930415

11.

N	-0.2779254	-1.4848949	1.9993780
C	-1.3369324	-2.1814819	1.4742720
N	-1.2782534	-2.2815309	0.1783500
C	-0.1151164	-1.6416859	-0.1696170
C	2.1652426	-0.1396929	-0.1380350
N	1.6359276	-0.3776269	1.0321470
C	0.5115926	-1.1203799	0.9532900
C	0.4285566	-1.3329999	-1.4619230
O	0.0157806	-1.5743239	-2.5674190
N	3.2563116	0.6742411	-0.2127290
N	1.6291266	-0.5765859	-1.3072520
H	-0.0748844	-1.3260059	2.9730600
H	-2.1131034	-2.5947159	2.1014850
H	3.7355216	0.7885001	0.6675110
H	3.8681566	0.5831381	-1.0080780
H	2.0703146	-0.3458889	-2.1868860

Ag	-1.4327384	1.2931423	0.0583683
C	1.3388596	3.0717043	-0.0360207
C	-4.5486664	0.4239753	-0.6136617
S	-3.0840344	-0.0398697	-1.0837777
S	0.3924766	2.4531923	1.1091803
H	2.2225916	3.6313003	0.2587803
H	1.1464586	2.9738103	-1.0993017
H	-5.4154794	-0.0743567	-1.0429617
H	-4.7397844	1.1990353	0.1218403

12.

N	-0.0598768	-1.5103157	1.9949940
C	-1.0561488	-2.3103517	1.4990740
N	-1.0166788	-2.4141597	0.2018120
C	0.0670002	-1.6625877	-0.1757100
C	2.1468512	0.1023573	-0.2103180
N	1.6877292	-0.1955377	0.9748190
C	0.6599852	-1.0704457	0.9288110
C	0.5303932	-1.2945427	-1.4826630
O	0.1115502	-1.5805917	-2.5752810
N	3.1200672	1.0542893	-0.3175170
N	1.6398882	-0.4049737	-1.3633590
H	0.1387122	-1.3128137	2.9623160
H	-1.7712778	-2.7966007	2.1461270
H	3.6089232	1.2248083	0.5485880
H	3.7192262	1.0318243	-1.1277230
H	2.0202952	-0.1203137	-2.2554560
C	0.9469176	3.1625637	-0.1263906
C	-4.4959234	-0.0494023	-0.3802756
S	-3.0126774	-0.3424173	-0.9243976
S	0.0755556	2.4720567	1.0381714
Au	-1.5351644	1.1287617	0.0745774
H	1.7535356	3.8293167	0.1641664
H	0.7681706	3.0130117	-1.1849456
H	-5.3091374	-0.6553153	-0.7732266
H	-4.7379164	0.7013797	0.3638074

13.

C	1.0943613	-0.9213963	-1.6870294
C	-0.3205526	-0.7985581	-1.7798481
N	-1.0742428	-1.9097595	-2.0089163
N	-0.9697580	0.3253965	-1.5622903
C	-0.2869816	1.4632156	-1.2904298
N	1.1076870	1.3856350	-1.3107293
C	1.7726922	0.2262401	-1.4446912
O	-0.8028598	2.5169936	-0.9385226
H	1.6090876	-1.8628138	-1.8111420

H	-2.0342000	-1.7245446	-2.2647366
H	-0.6404248	-2.6773013	-2.4974476
H	1.5995949	2.2467462	-1.1208781
H	2.8524765	0.2688137	-1.3724141
C	-0.9770877	-2.8426922	0.6426319
H	-0.8837920	-3.8864004	0.3204412
C	-0.5945756	2.2989727	1.3711271
H	-0.3412782	3.3614156	1.3982433
H	-1.6463648	2.0217957	1.2707865
H	-1.9688676	-2.3777584	0.6262232
O	0.2894569	1.4665926	1.5726603
O	0.0098466	-2.2547901	1.0509366
Cu	0.2160474	-0.4090755	1.3982020

14.

C	-1.1388962	-0.1293040	-2.1307049
C	0.2809319	-0.2486914	-2.1649411
N	1.0088734	0.6258901	-2.9105887
N	0.9545023	-1.1227380	-1.4474687
C	0.2980558	-2.0265239	-0.6794995
N	-1.1001695	-1.9867224	-0.7101453
C	-1.7918858	-1.0461790	-1.3759954
O	0.8386440	-2.8160787	0.0823066
H	-1.6753796	0.6255217	-2.6866616
H	1.9708738	0.3568938	-3.0610307
H	0.5552883	1.0662177	-3.6951452
H	-1.5733610	-2.6788640	-0.1477596
H	-2.8713638	-1.0795051	-1.2919761
C	0.9529410	2.8103694	-1.0701276
H	0.8772045	3.5423784	-1.8854939
C	0.6155813	-1.7733199	2.1668059
H	0.4142189	-2.7602810	2.5947587
H	1.6561881	-1.5188496	1.9461497
H	1.9416343	2.3880774	-0.8495271
O	-0.3010549	-0.9664256	2.0569319
O	-0.0358342	2.5276398	-0.4242438
Ag	-0.2918541	0.8793158	0.9595973

15.

C	-1.3495705	-0.0253918	-2.2281221
C	0.0751657	0.0046926	-2.2747709
N	0.7146469	1.0277057	-2.8545056
N	0.8277817	-0.9433406	-1.7331835
C	0.2518657	-1.9808241	-1.1310585
N	-1.1256592	-2.0774441	-1.1343484
C	-1.9083451	-1.1004129	-1.6266807
O	0.8766400	-2.8114141	-0.4345949

H	-1.9628732	0.7438109	-2.6761320
H	1.7154645	0.9590634	-2.9507066
H	0.2269452	1.6768492	-3.4455423
H	-1.5180713	-2.8662410	-0.6405689
H	-2.9783302	-1.2402220	-1.5384540
C	-1.5789561	3.1532005	-0.0373810
H	-1.7748467	4.2247052	0.0856460
C	1.0348752	-1.8993716	1.2698015
H	1.2022348	-2.8565323	1.7613417
H	1.9046327	-1.3468869	0.9160531
H	-1.8444385	2.6623634	-0.9817378
O	-0.0538739	-1.3168692	1.5318936
O	-1.0741193	2.5443940	0.8837007
Au	-0.5445794	0.5829034	1.0830279

16.

C	-1.7365460	-1.0422770	-1.1156614
C	-0.8469233	-0.3510138	-1.9971506
N	-1.3306742	0.6155206	-2.8153963
N	0.4503313	-0.5435829	-2.0232759
C	1.0281189	-1.5021356	-1.2319596
N	0.1315665	-2.3039272	-0.4843720
C	-1.1848894	-2.0555103	-0.4022858
O	2.2128772	-1.6866818	-1.1152279
H	-2.7984068	-0.8458898	-1.0850185
H	-0.7150410	0.8982645	-3.5632161
H	-2.3168528	0.6395383	-3.0152058
H	0.5538999	-3.0701074	0.0180285
H	-1.7702063	-2.7058894	0.2374091
C	-0.4155102	2.8148913	-1.2049690
H	-0.2372567	3.4728100	-2.0513261
C	0.7293947	-0.8678720	3.5657655
H	0.6462919	-1.4996435	4.4473408
H	1.7252679	-0.5583579	3.2635195
H	-1.4301573	2.7541537	-0.8303312
S	-0.6126131	-0.4401651	2.7833145
S	0.8360006	2.0315306	-0.5550025
Cu	-0.0010503	0.7732212	1.0523516

17.

C	-1.6217084	0.4805582	-1.8305200
C	-0.8986538	1.6513873	-1.4362463
N	-1.5712540	2.7244903	-0.9609418
N	0.4114545	1.7391414	-1.4562007
C	1.1715572	0.7042484	-1.9311485
N	0.4533254	-0.3815619	-2.4889409
C	-0.8808343	-0.5029939	-2.3980797

O	2.3747452	0.6448905	-1.8857098
H	-2.6984600	0.4133063	-1.7758816
H	-1.0492534	3.5862348	-0.9179106
H	-2.5595149	2.8032107	-1.1313062
H	1.0170589	-1.0902238	-2.9334135
H	-1.3230741	-1.4011321	-2.8136595
C	-0.7360239	2.3680432	1.9200159
H	-0.6332241	3.4179797	2.1838629
C	0.8970616	-3.6920232	0.2001488
H	0.9279306	-4.7504094	-0.0512244
H	1.8483787	-3.1778322	0.3029579
H	-1.7309215	1.9416481	1.9722592
S	-0.5320311	-2.9805941	0.4032734
S	0.5819715	1.5467799	1.5001032
Ag	-0.1221682	-0.6633261	0.8880591

18.

C	-1.7100418	-1.5722315	-1.6401631
C	-1.0623798	-0.5848955	-2.4436185
N	-1.7769917	0.4414767	-2.9605451
N	0.2307409	-0.5679470	-2.6797957
C	1.0426591	-1.5578704	-2.1878207
N	0.3866291	-2.6080594	-1.5084262
C	-0.9235732	-2.5909965	-1.2162218
O	2.2444810	-1.5709612	-2.2735066
H	-2.7706688	-1.5555926	-1.4368266
H	-1.3367247	0.9492350	-3.7119908
H	-2.7809987	0.3822154	-2.9845323
H	0.9803938	-3.3698677	-1.2172713
H	-1.3117991	-3.4330818	-0.6551765
C	-0.4762118	2.5554354	-1.2518985
H	-0.3477949	3.3198025	-2.0137311
C	0.7507484	-1.7561370	3.2243493
H	0.6167455	-2.4441759	4.0559481
H	1.7572412	-1.4192937	2.9987908
H	-1.4284874	2.5053679	-0.7399745
S	-0.5530200	-1.2922504	2.4029584
S	0.7686396	1.5797971	-0.9520298
Au	0.0839759	0.1311812	0.7140386

19.

C	0.5904828	-0.6266944	-1.7772353
C	0.2395230	1.5496585	-0.6318538
C	-1.1950261	1.3865836	-0.8204506
C	-1.6177468	0.2786566	-1.4624420
O	0.7619882	2.4478730	0.0002819
O	1.3156232	-1.5586881	-2.0557238

C	-2.1202520	2.4519111	-0.3357784
N	1.0227214	0.5230372	-1.1592308
N	-0.7654482	-0.6569034	-1.9722441
H	-2.6692375	0.0925593	-1.6474998
H	-2.2662640	2.4170989	0.7465027
H	-1.7140836	3.4363322	-0.5725083
H	-3.1015201	2.3575157	-0.8002102
H	2.0218035	0.5988213	-1.0144786
H	-1.1235681	-1.4774025	-2.4348578
C	0.1968047	1.6761050	2.3992343
H	1.0704627	2.2039935	2.7969165
C	0.3747011	-3.2124581	-0.1960341
H	0.1867685	-4.0426331	-0.8857699
H	1.4066485	-3.0207419	0.1141969
H	-0.7011061	2.2591001	2.1756124
O	-0.5682918	-2.5564477	0.2216021
O	0.2372252	0.4658201	2.2649321
Cu	-0.4900159	-0.9595608	1.2631378

20.

C	-0.5676252	-1.8240894	-1.2080396
C	0.5461743	0.2851310	-1.9161292
C	-0.7437055	0.9581460	-1.8697541
C	-1.8229493	0.2184270	-1.5335967
O	1.6062834	0.8358110	-2.1618264
O	-0.4971485	-2.9742181	-0.8609727
C	-0.8132630	2.3982819	-2.2478520
N	0.5251568	-1.0585806	-1.5710593
N	-1.7491903	-1.1129140	-1.2452824
H	-2.8187229	0.6452644	-1.4994869
H	-0.1947970	3.0194407	-1.5963771
H	-0.4345785	2.5418946	-3.2620842
H	-1.8373760	2.7688764	-2.2035154
H	1.4121458	-1.5459475	-1.5888637
H	-2.5779674	-1.6392679	-1.0188876
C	2.2918979	1.6425974	0.0698258
H	3.3318530	1.5176546	-0.2518747
C	-2.2539581	-1.6030690	2.8140831
H	-3.2673116	-2.0082097	2.9419627
H	-1.4541719	-1.9986443	3.4558817
H	1.7597106	2.5396628	-0.2647022
Ag	-0.2272857	0.2754649	1.3355247
O	-2.0520140	-0.7438458	1.9826292
O	1.7916558	0.8286234	0.8263753

21.

C	0.9968532	-1.2346470	-1.9588430
C	0.2651098	1.1310741	-2.0294288

C	-1.1145548	0.6901331	-2.1206292
C	-1.3400595	-0.6387182	-2.0992677
O	0.6106456	2.2976976	-1.8872257
O	1.8631645	-2.0570829	-1.7920730
C	-2.2029661	1.7026428	-2.2434613
N	1.2173138	0.1260437	-2.0125798
N	-0.3384457	-1.5621257	-2.0596236
H	-2.3427238	-1.0462772	-2.1575532
H	-2.3585056	2.2689779	-1.3222867
H	-1.9571027	2.4270778	-3.0215653
H	-3.1494226	1.2261164	-2.4981718
H	2.1828189	0.4173936	-1.9241586
H	-0.5403636	-2.5453240	-2.1452033
C	0.1688852	2.6608622	0.4006935
H	0.8535309	3.5082784	0.3265540
C	-0.0662287	-3.1267174	0.9665274
H	-0.4544472	-4.1327653	1.1643368
H	0.9305368	-3.0086420	0.5222194
H	-0.8607492	2.7837160	0.0645842
O	-0.7802018	-2.1755494	1.2437689
O	0.5649913	1.6437546	0.9645690
Au	-0.2440523	-0.2127994	1.0657350

22.

C	1.2728612	-0.2213761	-1.6211063
C	-0.0891326	1.7314272	-0.9116209
C	-1.2847414	1.0269754	-1.3536673
C	-1.1177012	-0.1703665	-1.9537323
O	-0.0858968	2.8269781	-0.3820202
O	2.3219388	-0.8189511	-1.6272250
C	-2.6075751	1.6987985	-1.2115918
N	1.1004506	1.0279622	-1.0639829
N	0.1072183	-0.7421729	-2.1360272
H	-1.9547753	-0.7284088	-2.3571791
H	-2.8784294	1.8543000	-0.1647421
H	-2.5804899	2.6864093	-1.6760254
H	-3.3987833	1.1133995	-1.6797408
H	1.9427431	1.4831930	-0.7356389
H	0.1996328	-1.6208406	-2.6198899
C	-0.5292534	2.1996893	2.0774933
H	-0.1908312	3.1605196	2.4554328
C	0.8061583	-3.3686548	-0.0696271
H	0.9388781	-4.4015003	-0.3851278
H	1.6442920	-2.6892472	-0.2108652
H	-1.4998512	2.1746169	1.6006705
S	-0.6154169	-2.9343848	0.5580659
S	0.3947692	0.8944418	2.3217431

Cu	-0.4088670	-0.8270406	1.1879675

23.

C	1.2085563	-2.0368374	-0.6141840
C	0.0985491	-0.5710685	-2.2835990
C	-1.1914739	-1.0814542	-1.8437127
C	-1.1937123	-2.0209942	-0.8777535
O	0.2517903	0.2951554	-3.1262082
O	2.2012685	-2.3808728	-0.0168980
C	-2.4277857	-0.5976710	-2.5220197
N	1.2018896	-1.1001648	-1.6238472
N	-0.0498602	-2.5024823	-0.3094358
H	-2.1124065	-2.4717151	-0.5207421
H	-2.6132593	0.4633320	-2.3384408
H	-2.3359585	-0.7146277	-3.6035573
H	-3.3033260	-1.1516801	-2.1837129
H	2.1090474	-0.7554034	-1.9108145
H	-0.0855983	-3.2458377	0.3691928
C	-0.1920866	2.4894728	-1.8599304
H	0.1848524	3.0324451	-2.7225151
C	0.8863029	-1.5327674	2.9119584
H	1.0059922	-2.2709914	3.7029431
H	1.6852213	-1.4623975	2.1766385
H	-1.1691116	2.0359502	-1.9573431
S	-0.4570809	-0.6422175	2.8942713
S	0.6869742	2.4701336	-0.5047046
Ag	-0.2294823	0.9166144	1.0755614

24.

C	0.4564909	-0.5504786	-3.2388907
C	0.2778528	1.6069178	-2.0246768
C	-1.1438575	1.3555486	-1.8273673
C	-1.6431310	0.1992579	-2.2997674
O	0.8873224	2.5413858	-1.5382181
O	1.1422875	-1.4093723	-3.7423023
C	-1.9678511	2.3880051	-1.1391479
N	0.9479561	0.6520248	-2.7807994
N	-0.8876188	-0.7084922	-2.9893428
H	-2.6888151	-0.0622350	-2.1870418
H	-1.6478593	2.5430712	-0.1066173
H	-1.8707397	3.3509621	-1.6448764
H	-3.0208788	2.1066709	-1.1278004
H	1.9326898	0.8154949	-2.9446489
H	-1.2989451	-1.5530734	-3.3537457
C	0.4924126	2.6556545	1.6158378
H	0.6049080	3.6089896	2.1286066
C	1.1852803	-2.5584348	-0.8645755

H	1.3269544	-3.4284666	-1.5005446
H	1.9952269	-1.8395541	-0.8093907
H	0.7942347	2.5978958	0.5691584
S	-0.2055629	-2.4159444	-0.0625911
S	-0.1396873	1.4402154	2.4674395
Au	-0.1954261	-0.4507479	1.1516667

25.

C	-0.2476836	-1.0221548	-1.6498941
C	1.8121594	0.3903432	-0.3279081
N	1.7230674	-0.9794108	-0.3412151
C	0.7177544	-1.6581648	-0.9652021
C	-0.2402866	0.4248312	-1.7230641
O	-1.1036266	1.1187642	-2.2122091
N	0.8591934	1.0158402	-1.0844561
O	2.6219264	0.9838972	0.3592559
H	-1.0325736	-1.5615838	-2.1581601
H	2.4301144	-1.4680978	0.1857219
H	0.7588894	-2.7369418	-0.8793881
H	0.9074384	2.0264052	-1.1122731
C	-3.4371024	0.1395915	-0.0537054
C	1.4254406	0.8042175	2.5812376
O	0.6058216	-0.0897385	2.4167466
O	-2.8592954	-0.2403275	0.9494736
Cu	-1.1127874	-0.1697705	1.6487686
H	-4.5174274	-0.0369295	-0.1323714
H	-2.9118494	0.6428575	-0.8776604
H	2.4004246	0.5642395	3.0148796
H	1.2004026	1.8521325	2.3614236

26.

C	-1.1500795	0.3504280	-1.5208021
C	0.4198045	-1.6761910	-0.3250661
N	-0.9555325	-1.6480690	-0.2702041
C	-1.7066485	-0.6569170	-0.8240431
C	0.2884805	0.4010470	-1.6834691
O	0.9121495	1.2971500	-2.2115641
N	0.9623585	-0.6665480	-1.0803771
O	1.0783095	-2.4850290	0.2929299
H	-1.7533395	1.1129030	-1.9900051
H	-1.3864465	-2.3914190	0.2569329
H	-2.7769475	-0.7460960	-0.6832211
H	1.9716405	-0.6672420	-1.1594951
C	0.4074948	3.3156602	-0.4564205
C	0.6632118	-1.6682658	2.7851535
Ag	-0.1346132	1.1413902	1.7660045
O	-0.2294972	-0.8525908	2.6350585

O	0.6346928	2.8113952	0.6250735
H	1.1609658	3.9607502	-0.9250505
H	-0.5451112	3.1758102	-0.9810405
H	0.4175138	-2.6889478	3.1025405
H	1.7215928	-1.4192178	2.6470645

27.

C	0.5380879	-1.8232669	-0.6268539
C	-0.2509511	0.6365401	-1.7628589
N	1.0517469	0.2137571	-1.7133549
C	1.4327989	-0.9682339	-1.1471259
C	-0.8663501	-1.4729019	-0.6245849
O	-1.7483901	-2.0676239	-0.0449909
N	-1.1533831	-0.2826629	-1.3086289
O	-0.5546641	1.7685771	-2.1025189
H	0.8380629	-2.7622829	-0.1867559
H	1.7365679	0.8611971	-2.0711329
H	2.4969679	-1.1680849	-1.1555059
H	-2.1240311	0.0033411	-1.3243389
C	0.0371786	3.0682084	-0.0838273
C	-0.5600114	-1.5563156	3.0880947
O	0.1643846	-0.5805256	3.2191537
O	0.8572236	2.4088094	0.5527117
Au	0.4864876	0.9268724	1.8946047
H	0.4107636	3.8107894	-0.7918493
H	-1.0387114	2.9959244	0.0858427
H	-0.6129254	-2.2597146	3.9271787
H	-1.1408524	-1.7524036	2.1767417

28.

C	1.1225183	1.4857258	-1.4787745
C	-0.6620948	-0.6396917	-2.0271537
N	-1.0142670	0.6902639	-2.1064331
C	-0.1611914	1.7089468	-1.8080795
C	1.6267607	0.1271708	-1.4275003
O	2.7464069	-0.2019305	-1.1047472
N	0.6582586	-0.8422069	-1.7125611
O	-1.4687856	-1.5321411	-2.1682090
H	1.8062410	2.3014589	-1.2970950
H	-1.9643509	0.8722137	-2.3895953
H	-0.5782088	2.7058293	-1.8833793
H	0.9619584	-1.8071624	-1.6807227
C	2.6013260	0.6584652	1.5821253
H	3.6272372	0.4345133	1.8630052
C	-2.8805896	-1.2450746	0.1371693
H	-3.8345425	-1.5033834	-0.3151424
H	-2.1608853	-2.0457758	0.2572258

H	2.4439155	1.4695685	0.8827966
S	-2.6314747	0.2778913	0.6249631
S	1.4087523	-0.2031723	2.2422301
Cu	-0.5405114	0.3298831	1.3427514

29.

C	-1.5647112	-1.5228626	1.3662611
C	0.5874698	0.0255832	2.3543849
N	-0.7353507	0.3913237	2.4806264
C	-1.7662918	-0.3437991	1.9789184
C	-0.2165300	-2.0368171	1.2203141
O	0.0958075	-3.0571515	0.6481403
N	0.7668816	-1.1923819	1.7489368
O	1.4951221	0.7478728	2.7040784
H	-2.3913080	-2.1235813	1.0173083
H	-0.9028202	1.2529590	2.9758615
H	-2.7548455	0.0717822	2.1318899
H	1.7248004	-1.5085464	1.6673161
C	-0.7365855	-2.4035658	-2.0020197
H	-0.5745127	-3.3693263	-2.4748218
C	1.3308615	2.7740979	0.9068914
H	1.5858855	3.5531438	1.6212641
H	2.1233091	2.0905427	0.6267593
H	-1.5175994	-2.3456930	-1.2544653
S	-0.1675823	2.7288220	0.3021458
S	0.1670942	-1.1522222	-2.4607148
Ag	-0.2780000	0.7538027	-1.0634779

30.

C	-1.6371095	-0.7167969	-2.0590328
C	0.8247205	-2.1117385	-1.8470233
N	-0.3623092	-2.5525060	-1.2853631
C	-1.5328012	-1.8714025	-1.3716849
C	-0.4727656	-0.1866037	-2.7176994
O	-0.4203660	0.8869311	-3.3109587
N	0.6736773	-0.9429731	-2.5720371
O	1.8723782	-2.6788149	-1.6982935
H	-2.5723501	-0.1850324	-2.1432687
H	-0.3052668	-3.4371374	-0.8055696
H	-2.3797739	-2.3247785	-0.8706060
H	1.5111157	-0.6010462	-3.0257536
C	0.0913597	2.4146257	-1.6506517
H	0.3784080	3.0923578	-2.4481461
C	0.7138659	-2.0914143	2.8540686
H	0.4918163	-2.8573135	3.5937651
H	1.7462545	-1.9673533	2.5435109
H	-0.9642656	2.3066805	-1.4424523

S	-0.5044605	-1.2025803	2.2907585
S	1.2720673	1.8027749	-0.7095695
Au	0.3180026	0.3047791	0.7649722

Additional QTAIM, NCIplot and NBO analyses

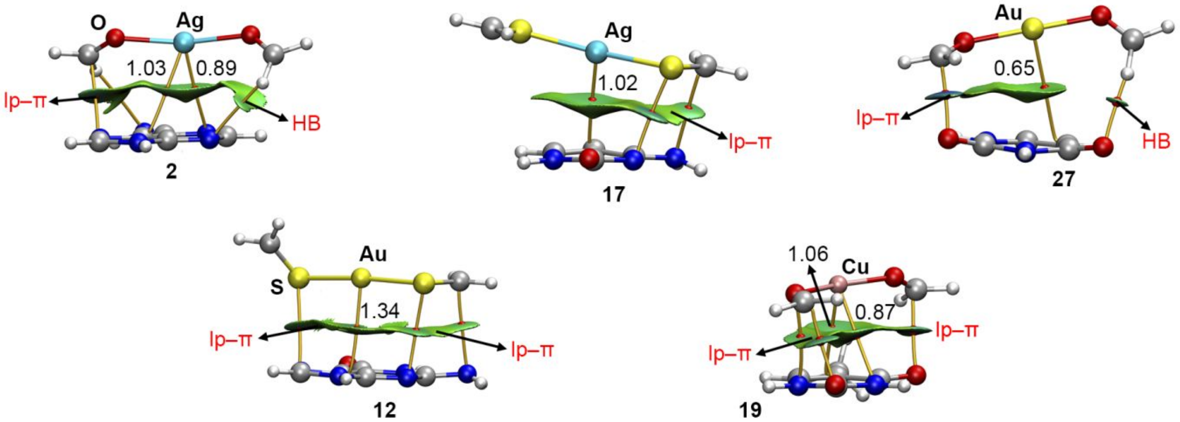

Figure S1. NCIplot analysis and AIM distribution of intermolecular bond critical points (BCP in red spheres) and bond paths in complexes **2**, **12**, **17**, **19** and **27**. The value of density at the BCPs characterizing the Rg-π interaction is also indicated. Ancillary interactions are highlighted in red. NCIplot surfaces involving only intermolecular contacts between the Rg coordination complex and the nuclebases are also indicated. NCIplot colour range –0.035 a.u. ≤ $(\text{sign}\lambda_2)\rho$ ≤ +0.035 a.u. Isosurface value |RGD| = 0.5 and ρ cut-off 0.04 a.u.

Cartesian coordinates of the selected PDB structures

2OIJ

N	2.4886747	-4.1550141	0.4216181
C	1.8956747	-3.0060141	-0.1293819
O	2.6306747	-2.0750141	-0.5113819
N	0.5446747	-2.9420141	-0.2203819
C	-0.2013253	-3.9600141	0.2226181
N	-1.5283253	-3.8760141	0.0966181
C	0.3786747	-5.1240141	0.8116181
C	1.7136747	-5.1780141	0.8856181
N	2.0296747	0.2209859	2.6246181
C	1.4306747	-1.0010141	2.8216181
N	0.1596747	-1.0050141	2.5106181
C	-0.0943253	0.2919859	2.0896181
C	-1.2863253	0.8889859	1.6446181
O	-2.4053253	0.3819859	1.5286181
N	-1.0973253	2.2279859	1.3176181
C	0.0796747	2.9109859	1.4176181
N	0.0506747	4.1899859	1.0286181
N	1.2046747	2.3759859	1.8546181
C	1.0476747	1.0659859	2.1646181
N	-1.6063253	3.6289859	-3.3533819
C	-2.9373253	3.3119859	-3.5503819
N	-3.2773253	2.1849859	-2.9863819
C	-2.1113253	1.7659859	-2.3783819
C	-1.8233253	0.6189859	-1.6643819
O	-2.5393253	-0.3670141	-1.4243819
N	-0.5223253	0.6369859	-1.2263819
C	0.4246747	1.5879859	-1.4853819
N	1.6656747	1.3619859	-1.0113819
N	0.1796747	2.6499859	-2.1623819
C	-1.0943253	2.6779859	-2.5773819
Au	0.5736747	-0.6010141	-0.8743819
H	3.5043995	-4.1899131	0.4403661
H	2.2469562	-6.0330749	1.3083991
H	-0.2478169	-5.9387232	1.1719341
H	-1.9624975	-3.0344559	-0.2765775
H	-2.1343953	-4.6083432	0.4444818
H	-1.9561213	2.7047629	1.0492911
H	0.8712833	4.7625028	1.1995681
H	-0.8120565	4.6665326	0.7988643
H	2.9820286	0.4866470	2.8580525
H	1.9875631	-1.8536492	3.2135842
H	2.4035009	1.3098169	-1.7175606
H	1.9653682	1.9325247	-0.2091390

H	-1.1179474	4.4453986	-3.7093614
H	-3.6008139	3.9535696	-4.1333872

2XY5

C	1.3310212	2.1463856	-5.6012549
C	1.8280212	1.5303856	-4.4612549
C	0.1220212	2.8203856	-5.5642549
C	-0.6049788	2.8803856	-4.3992549
C	-0.1159788	2.2683856	-3.2692549
C	-1.0139788	2.4153856	-2.0822549
C	1.1020212	1.5853856	-3.2782549
O	1.6030212	0.9943856	-2.2252549
N	-1.2249788	-2.8956144	2.8827451
C	-2.3039788	-2.1156144	3.1687451
N	-2.8359788	-1.5276144	2.1197451
C	-2.0259788	-1.9406144	1.0767451
C	-2.0459788	-1.6686144	-0.2982549
N	-2.9469788	-0.8896144	-0.9002549
N	-1.0999788	-2.2516144	-1.0512549
C	-0.1979788	-3.0466144	-0.4612549
N	-0.0819788	-3.3816144	0.8127451
C	-1.0329788	-2.7796144	1.5317451
C	3.7140212	-2.2486144	2.6337451
C	3.3870212	-1.7096144	1.3907451
C	2.9940212	-1.9036144	3.7677451
C	1.9540212	-1.0026144	3.6667451
C	1.6270212	-0.4656144	2.4427451
C	0.4840212	0.4953856	2.5007451
C	2.3400212	-0.7996144	1.2887451
O	2.0720212	-0.2806144	0.1187451
Cu	0.7110212	0.7883856	-0.4332549
N	-0.8629788	2.0063856	-0.5392549
C	-1.8539788	2.5113856	0.4177451
C	-1.4309788	2.0183856	1.7787451
N	-0.2049788	1.2383856	1.5977451
H	3.9313900	-1.9660239	0.4813838
H	4.5427035	-2.9581614	2.7092892
H	3.2589096	-2.3312185	4.7375594
H	1.3886047	-0.7135981	4.5569699
H	2.7750965	0.9903145	-4.4505013
H	1.9000558	2.0979676	-6.5336583
H	-0.2575103	3.3075482	-6.4656141
H	-1.5599615	3.4103188	-4.3736890
H	-1.8932568	3.6128975	0.3833526
H	-2.8632369	2.1404821	0.1647814
H	-1.2468075	2.8549066	2.4743834
H	-2.2029083	1.3711051	2.2298910

H	0.5492812	-3.4734105	-1.1406495
H	-2.6802078	-2.0156958	4.1879902
H	-0.6253728	-3.4047537	3.5185727
H	-1.9258850	2.9992348	-2.2675510
H	0.1074698	0.5673225	3.5403652
H	-2.8634101	-0.7348842	-1.8944912
H	-3.6871376	-0.4596186	-0.3678709

5CCW

N	-2.8005148	-2.2328338	-1.3690177
C	-3.6935148	-2.1128338	-0.3360177
N	-3.1285148	-2.1528338	0.8369823
C	-1.7805148	-2.3188338	0.5619823
C	-0.6725148	-2.4478338	1.4349823
O	-0.6575148	-2.4398338	2.6649823
N	0.5154852	-2.5978338	0.7319823
C	0.6254852	-2.6188338	-0.6310177
N	1.8614852	-2.7608338	-1.1170177
N	-0.3985148	-2.5178338	-1.4560177
C	-1.5625148	-2.3708338	-0.7960177
O	-7.3335148	1.9351662	-0.9830177
C	-6.2615148	1.7531662	-0.4260177
N	-6.2315148	1.7391662	0.9579823
C	-7.4635148	1.9331662	1.7179823
C	-5.0195148	1.5381662	1.6149823
O	-4.9995148	1.5251662	2.8309823
C	-3.8405148	1.3551662	0.8889823
N	-2.5205148	1.1341662	1.2909823
C	-2.0775148	1.0511662	2.6779823
N	-5.0655148	1.5731662	-1.1420177
C	-5.1195148	1.6051662	-2.5890177
C	-3.8425148	1.3731662	-0.4630177
N	-2.4995148	1.1661662	-0.8440177
C	-1.9455148	1.0931662	-2.1960177
C	-1.7575148	1.0441662	0.2229823
Au	0.2114852	0.7291662	0.2209823
C	2.2184852	0.4031662	0.2119823
N	2.9764852	0.2621662	1.2699823
C	4.3004852	0.0661662	0.8939823
C	2.5164852	0.3321662	2.6399823
N	3.0054852	0.2751662	-0.8500177
C	2.5824852	0.3441662	-2.2390177
C	4.3184852	0.0651662	-0.4410177
N	5.5274852	-0.1378338	-1.1270177
C	5.5734852	-0.1548338	-2.5790177
C	6.7214852	-0.3198338	-0.4300177
O	7.7724852	-0.4888338	-1.0430177

N	6.7024852	-0.3178338	0.9649823
C	7.9424852	-0.5258338	1.7029823
C	5.4874852	-0.1228338	1.6349823
O	5.4594852	-0.1288338	2.8559823
H	-1.1938205	1.6877427	2.8107685
H	-2.8987836	1.3866991	3.3183425
H	-1.8323070	0.0050135	2.9167999
H	-7.2352921	1.7118292	2.7650835
H	-7.8096991	2.9705540	1.6121566
H	-8.2371626	1.2616882	1.3263309
H	-4.7186802	0.6756372	-3.0189875
H	-6.1766939	1.6955318	-2.8682521
H	-4.5820607	2.4764818	-2.9932773
H	8.6490809	0.2837107	1.4768461
H	7.6884278	-0.5312407	2.7678367
H	8.3934257	-1.4801551	1.4012119
H	6.6266540	-0.2702034	-2.8598675
H	5.0113268	-1.0059885	-2.9870027
H	5.2009822	0.7876380	-2.9992297
H	-1.0017518	1.6510213	-2.2177305
H	-1.7548500	0.0463254	-2.4698110
H	-2.6324272	1.5480662	-2.9123660
H	2.8700349	-0.5666828	-2.7727485
H	1.4880288	0.4137685	-2.2484465
H	3.0015226	1.2288719	-2.7340352
H	3.3486588	0.0633278	3.2989572
H	2.1878352	1.3543369	2.8727702
H	1.6733922	-0.3577113	2.7887680
H	1.3349477	-2.7067607	1.3211268
H	2.5712368	-3.1936161	-0.5399878
H	1.8987133	-3.0201378	-2.0959529
H	-4.7645887	-2.0075446	-0.5156233
H	-3.0091905	-2.3434008	-2.3536630

6M2P

N	2.4481335	-1.5024002	-0.2053452
C	2.5721335	-1.4344002	1.1506548
N	1.4861335	-1.0204002	1.7596548
C	0.6041335	-0.7714002	0.7166548
C	-0.7168665	-0.2844002	0.6796548
N	-1.4058665	0.0535998	1.7686548
N	-1.2968665	-0.1394002	-0.5333452
C	-0.6118665	-0.4894002	-1.6263452
N	0.6291335	-0.9624002	-1.7143452
C	1.1841335	-1.0764002	-0.4973452
H	3.4949651	-1.7222155	1.6559683
H	-2.1813928	0.7010323	1.6552058

H	-0.9054362	0.0890165	2.6478729
H	-1.1403126	-0.3455178	-2.5743068
Ag	-0.2498665	2.6605998	-0.7793452
Ag	-2.1528665	3.7265998	0.8086548
Ag	-2.8828665	1.7385998	-1.0003452
Ag	-1.7648665	3.0755998	-3.0793452
H	3.1438217	-1.7896686	-0.8815565

7ECL

N	-2.7251463	-4.0203212	-2.0521835
C	-2.2321463	-2.8083212	-1.6111835
O	-2.9601463	-1.9073212	-1.2081835
N	-0.8611463	-2.7003212	-1.6601835
C	0.0448537	-3.6503212	-2.0881835
O	1.2468537	-3.3953212	-2.0651835
C	-0.5371463	-4.8763212	-2.5341835
C	-1.8711463	-5.0043212	-2.5081835
N	0.9918537	5.0076788	-0.1361835
C	2.3298537	4.7946788	0.0998165
N	2.7088537	3.5506788	-0.0951835
C	1.5308537	2.9136788	-0.4591835
C	1.2248537	1.5926788	-0.7981835
N	2.1228537	0.6186788	-0.8251835
N	-0.0491463	1.2886788	-1.1161835
C	-0.9621463	2.2616788	-1.0891835
N	-0.8051463	3.5406788	-0.7841835
C	0.4738537	3.7966788	-0.4791835
N	-1.3291463	1.8296788	2.7328165
C	-0.1041463	2.3796788	3.0128165
N	0.9108537	1.5736788	2.7868165
C	0.3178537	0.4066788	2.3248165
C	0.8478537	-0.8293212	1.9128165
N	2.1538537	-1.1153212	1.8898165
N	-0.0161463	-1.7853212	1.5168165
C	-1.3211463	-1.5163212	1.5308165
N	-1.9391463	-0.3933212	1.8968165
C	-1.0581463	0.5466788	2.2918165
Ag	0.3048537	-1.1193212	-1.2171835
H	0.0958062	-5.6845343	-2.8992556
H	-2.3672010	-5.9184941	-2.8455210
H	2.9925426	5.6064465	0.4020923
H	3.0621689	0.8677613	-0.5339456
H	-1.9767068	1.9370380	-1.3466261
H	-0.0110511	3.4005669	3.3855743
H	2.8237549	-0.4537996	2.2509883
H	-1.9750142	-2.3164311	1.1724362
H	-2.2453350	2.2502258	2.8078396

H	0.4753457	5.8741831	-0.0613353
H	2.1152016	-0.0118210	-1.6305697
H	-3.7281871	-4.1501227	-2.0455320
H	2.4353683	-2.0500020	1.6286475

7EDV

N	3.0678466	1.1659892	-3.1598391
C	1.7478466	1.1779892	-2.7158391
O	1.0448466	2.1809892	-2.9178391
N	1.2528466	0.0889892	-2.1068391
C	2.0028466	-0.9730108	-1.9008391
N	1.4258466	-2.0090108	-1.2858391
C	3.3768466	-1.0230108	-2.3178391
C	3.8668466	0.0729892	-2.9378391
N	1.4218466	2.6119892	0.9391609
C	0.4418466	1.7709892	1.4871609
O	-0.6841534	2.2179892	1.6871609
N	0.7748466	0.4999892	1.8201609
C	2.0168466	0.0599892	1.6131609
N	2.3048466	-1.1950108	1.9621609
C	3.0188466	0.8929892	1.0331609
C	2.6768466	2.1459892	0.7151609
N	-3.8001534	1.6419892	0.3721609
C	-2.7031534	1.8129892	-0.4128391
N	-2.0071534	0.7419892	-0.5518391
C	-2.6691534	-0.2120108	0.1891609
C	-2.3621534	-1.5700108	0.3971609
O	-1.4031534	-2.2000108	-0.0368391
N	-3.3091534	-2.2020108	1.2131609
C	-4.4011534	-1.5660108	1.7551609
N	-5.2051534	-2.3090108	2.5341609
N	-4.6841534	-0.2820108	1.5611609
C	-3.7831534	0.3219892	0.7691609
Au	-0.4781534	0.4819892	-1.3868391
H	3.2611305	-1.5104579	2.0249307
H	1.5932762	-1.7078007	2.4677862
H	4.0350704	0.5368161	0.8632038
H	3.3855145	2.8527430	0.2767719
H	1.1387301	3.5546223	0.6997450
H	1.9596621	-2.8413565	-1.0820284
H	0.4602462	-1.9584923	-0.9370629
H	3.9882111	-1.9059426	-2.1440772
H	3.3895926	1.9964298	-3.6442914
H	-2.4526397	2.7767064	-0.8508708
H	-4.4614582	2.3532056	0.6584871
H	-5.0482228	-3.2884044	2.7158495
H	-6.0121484	-1.8574774	2.9431937

H	-3.1294470	-3.1847755	1.3918917
H	4.8951948	0.1378801	-3.2999123

7SDH

N	-1.2879017	-4.2716503	-0.3704867
C	-1.0539017	-4.6376503	0.8785133
C	0.1770983	-2.6796503	1.1755133
O	0.9760983	-1.8646503	1.9995133
N	-0.0879017	-2.3766503	-0.0784867
C	-0.8129017	-3.1296503	-0.8484867
N	-1.0629017	-2.6746503	-2.2154867
C	-0.2849017	-3.8556503	1.7025133
N	2.2990983	2.3753497	-0.5854867
C	2.9410983	2.8513497	0.4655133
C	2.3620983	0.8983497	1.6485133
O	2.4140983	0.1013497	2.8245133
N	1.7580983	0.4923497	0.5475133
C	1.7090983	1.1973497	-0.5644867
N	1.0150983	0.6863497	-1.7544867
C	2.9940983	2.1193497	1.6305133
N	-1.5749017	2.7033497	-2.0134867
C	-0.9199017	3.6563497	-1.3094867
N	-0.6139017	3.3053497	-0.0994867
C	-1.1279017	2.0353497	0.0075133
C	-1.1289017	1.1163497	1.0595133
N	-0.5789017	1.3813497	2.2445133
N	-1.7159017	-0.0866503	0.8505133
C	-2.2689017	-0.3306503	-0.3504867
N	-2.3109017	0.4633497	-1.4264867
C	-1.7219017	1.6423497	-1.1734867
Ag	0.6510983	-0.8386503	0.1145133
H	-1.4874015	-5.5876801	1.1960672
H	-1.1269252	-1.6582092	-2.2638032
H	-1.8159831	-3.1428485	-2.7088557
H	3.4047843	3.8325506	0.3573913
H	1.2850998	-0.2792970	-1.9424666
H	1.1811086	1.2771545	-2.5754260
H	-1.9634372	2.7815046	-2.9454941
H	-0.6970498	4.6309957	-1.7489215
H	-0.1114543	2.2657489	2.3866855
H	-2.7652895	-1.3024354	-0.4469163
H	-0.3775087	0.6213102	2.8877098
H	2.2532620	2.9448387	-1.4232386
H	-1.8585795	-4.8665394	-0.9639880
H	3.5208801	2.4762412	2.5132352
H	-0.0632397	-4.1315101	2.7309956

7SMB

N	-1.7877179	-5.0612564	-0.8316923
C	-1.2347179	-3.7632564	-1.2976923
O	-1.4317179	-3.4252564	-2.4106923
N	-0.4427179	-2.8752564	-0.3966923
C	-0.1827179	-3.2592564	0.9763077
O	0.4472821	-2.5542564	1.6913077
C	-0.7097179	-4.5652564	1.4593077
C	-1.5087179	-5.4352564	0.5423077
N	2.7012821	3.0977436	-1.7626923
C	2.1052821	1.7527436	-1.5126923
O	1.6032821	1.1507436	-2.3996923
N	2.1362821	1.1487436	-0.1526923
C	2.7472821	1.8747436	0.9503077
O	2.7772821	1.3937436	2.0303077
C	3.3442821	3.2227436	0.7193077
C	3.3072821	3.8157436	-0.6526923
N	-1.9667179	2.3797436	-1.7646923
C	-1.0857179	3.2697436	-1.2136923
N	-0.6367179	2.9077436	-0.0296923
C	-1.2517179	1.6847436	0.2053077
C	-1.1857179	0.7787436	1.2933077
N	-0.4367179	0.9847436	2.3843077
N	-1.9227179	-0.3492564	1.2103077
C	-2.6677179	-0.5492564	0.1153077
N	-2.8057179	0.2297436	-0.9636923
C	-2.0627179	1.3397436	-0.8556923
Ag	2.6202821	-0.9652564	2.6583077
Ag	0.8232821	-0.6782564	-1.3046923
H	0.2864474	1.6904508	2.3750756
H	-0.3864113	0.2180065	3.0414419
H	-2.4149657	2.4128538	-2.6699788
H	-0.8068419	4.1910717	-1.7264749
H	-3.2314099	-1.4878972	0.1007493
H	3.7988182	3.7753046	1.5400274
H	3.7307442	4.7940938	-0.8856620
H	2.6812133	3.4671589	-2.7046874
H	-1.9117966	-6.3955496	0.8718952
H	-0.5408497	-4.8895436	2.4855367
H	-2.3088382	-5.6314388	-1.4835202
